# Synthesis of an *o*‑Benzoquinone Arsenic Mononitride (As≡N) Complex and Its Reaction to Singlet Arsinonitrene

**DOI:** 10.1021/jacs.5c20377

**Published:** 2026-03-10

**Authors:** Weiyu Qian, Maria Eugenia Sandoval-Salinas, Rachel Crespo-Otero, Peter R. Schreiner, Artur Mardyukov

**Affiliations:** † Institute of Organic Chemistry, Justus Liebig University, Heinrich-Buff-Ring 17, Giessen 35392, Germany; ‡ Department of Chemistry, 4919 University College London, London WC1H 0AJ, U.K.

## Abstract

The generation and stabilization of heavier analogues of dinitrogen (N_2_) remain fundamental challenges in modern inorganic and materials chemistry. In marked contrast to N_2_, these species exhibit extremely high reactivities and transient lifetimes, making their synthesis, characterization, and utilization difficult. In this work, we introduce an efficient approach for the generation of an elusive *o*-benzoquinone—arsenic mononitride (As≡N) complex formed when *ortho*-phenyldioxoarsinoazide was exposed to UV or green light irradiation. Its recombination to arsinonitrene was observed upon irradiation with red light. The experimental data are well supported by density functional theory (DFT) and multireference (MS)-CASPT2­(10,10)-SOC/ANO-S-VDZP electronic structure computations. DFT analyses suggest that this strategy can be extended to heavier dipnictogen systems. Our findings enhance the fundamental understanding of heavier dipnictogen chemistry and establish a versatile synthetic platform for their synthesis and subsequent practical use.

## Graphical abstract

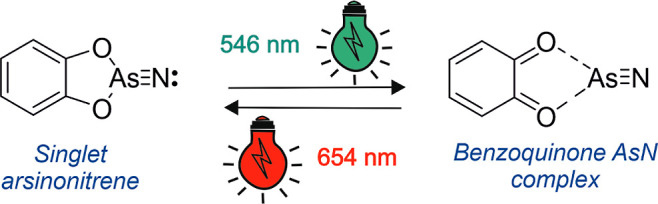

## Introduction

In stark contrast to exceptionally stable N_2_, the heavier dipnictogens are highly reactive transient species under ambient conditions (Table 1). While the only naturally occurring nitrogen allotrope is diatomic dinitrogen (the allotrope *C*_2*h*_-N_6_ has just recently been reported),^1^ phosphorus adopts a most favorable tetrahedral P_4_ structure (white phosphorus). Upon heating above 1100 K, P_4_ dissociates into two P_2_ molecules.^2^ In contrast to the exceptionally strong triple bond in N_2_ (*D*_0_ = 224.9 kcal mol^–1^),^3^ due to orbital mismatches, the bond dissociation energies of heavier dipnictogens are much lower (for example, *D*_0_ = 146.6 ± 5.0 kcal mol^–1^ for PN),^4^ resulting in their thermodynamic instability. The pronounced tendency of heavier dipnictogens to oligomerize and polymerize under ambient conditions presents a significant challenge for their selective synthesis. Their kinetic lability can be mitigated through stabilization with *N*-heterocyclic carbenes (NHCs) or transition metal complexes, which effectively prevent dimerization or oligomerization.^5–11^ Recently, Mo et al. reported a plumbylone-promoted degradation of P_4_, leading to the formation of diphosphene lead complexes.^12^ In 2021, Cummins et al. described the transfer of PN from an anthracene precursor to an iron complex in solution; the intermediacy of PN was supported by its mass spectrometric identification.^13^ In 2023, we reported the selective preparation of PN through the pyrolysis of *ortho*-phenyldioxophosphino azide in the gas phase and discovered its photochemical equilibration with *ortho*-benzoquinone.^14^

**Table 1:** All Possible Dipnictogenes and Their Detection and/or Synthesis

species	detected	isolated
N_2_	√^a^	√^b^
PN	√^c^	√^15^
AsN	√^16^	this work
SbN	√ ^17,18^	
BiN	√^19^	
P_2_	√ ^20,21^	√^2^
AsP	√^22^	√^7^
SbP	√^23^	
BiP	√ ^19,24^	
As_2_	√ ^25,26^	√^27^
AsSb	√ ^24,28^	
AsBi	√^24^	
Sb_2_	√^23^	
SbBi	√^24^	
Bi_2_	√^29^	

a C. W. Scheele 1771.

b D. Rutherford 1772.

c Detected in star-forming regions.^30^

2D materials composed of pnictogen elements (Pn), where Pn encompasses N, P, As, Sb, and Bi, demonstrate outstanding performance in batteries, transistors, and photovoltaic materials.^31–34^ Specifically, these materials resolve challenges related to the absence of a band gap in, e.g., graphene, which have impeded the advancement of electronic microdevices.^34–38^ AsN, the heavier congener of PN, forms a distinct polymer (AsN)_*n*_ that qualifies as a novel semiconductor material.^39^

(AsN)_*n*_ possesses exceptional electronic and optical properties, showcasing promising applications in field-effect transistors (FETs).^33,39^ A recent study by Ceppatelli et al. reported the synthesis of crystalline singly bonded (AsN)_n_ from elemental As and N_2_ under a pressure of 20 GPa.^40^ These findings suggest that (AsN)_*n*_ monolayers, similar to other two-dimensional pnictogens,^31,32,39^ exhibit high hole mobility, unique anisotropic characteristics, and ambipolar transport behavior dominated by holes. These properties render (AsN)_*n*_ a promising candidate for the development of next-generation semiconductor materials.^40^ However, the facile synthesis and spectroscopic characterization of its monomer have remained challenging, leaving the molecule poorly understood. To date, the only method for synthesizing AsN monomers involves the gas-phase microwave discharge of mixtures of AsCl_3_ and N_2_, with characterization achieved through rotational spectroscopy.^16^ Subsequently, Perdigon and Femelat conducted a thorough rotational analysis.^41^

To the best of our knowledge, there are no reports concerning the selective preparation of AsN from a readily available and scalable molecular precursor. Existing methods require either high-temperature/high-pressure conditions or high-energy gas-phase microwave discharges.^40,41^ The higher congener of AsN, arsenic monophosphide (AsP), was synthesized via laser ablation of arsenic in the presence of PH_3_ and was studied in a neon matrix using time-resolved, laser-induced fluorescence spectroscopy.^22,42–44^ Cummins and co-workers demonstrated the activation of As_4_ to generate reactive AsP species, enabling the synthesis and characterization of novel arsenic–phosphorus complexes through trapping and functionalization reactions.^7^

Compounds bearing arsenic and nitrogen multiple bonds are rare, with only a few reported examples. Schulz and Villinger documented the synthesis of a binary arsenic–nitrogen five-membered heterocycle, tetrazarsole (A),^45^ and an arsa-diazonium salt featuring a formal arsenic–nitrogen triple bond (B) (Figure 1).^46^ They also reported the synthesis of reactive four-membered arsenic–nitrogen biradicaloid (C) and outlined its subsequent reaction with CS_2_, S_8_, and Se.^47^ Recently, Bockfeld and Tamm reported the synthesis of a formal stable carbene adduct of arsenic mononitride NHC=As–N=NHC (D) (NHC; *N*-heterocyclic carbene).^48^

**Figure 1:**
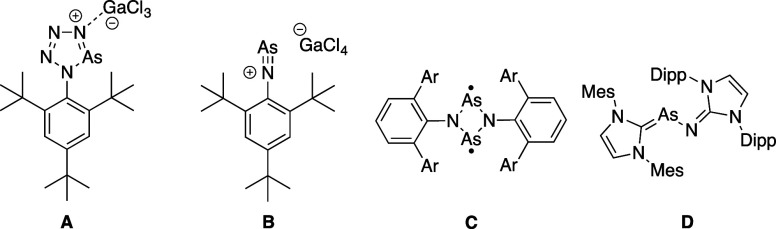
Structure of tetrazarsole (A), arsa diazonium (B), arsenic nitrogen biradicaloid (C), and a carbene adduct of arsenic mononitride (D) featuring AsN moieties.

Here, we report the novel *ortho*-benzoquinone-AsN (**3**-AsN) complex that forms upon the 546 nm irradiation of **1**. Subsequent red light irradiation prompts the recombination of the **3**-AsN complex, resulting in the formation of novel *ortho*-phenyldioxoarsino nitrene (**2**) (Scheme 1). Compound **2** displays a singlet ground state, as confirmed by comparison of experimental and computed infrared spectra including ^15^N-isotope labeling experiments. Although DFT results using a variety of modern functionals suggest a triplet ground state, multiconfiguration computations (for details, see below and Supporting Information) reveal the stabilization of the closed-shell singlet state over the triplet state, aligning well with our experimental findings.

**Scheme 1:**
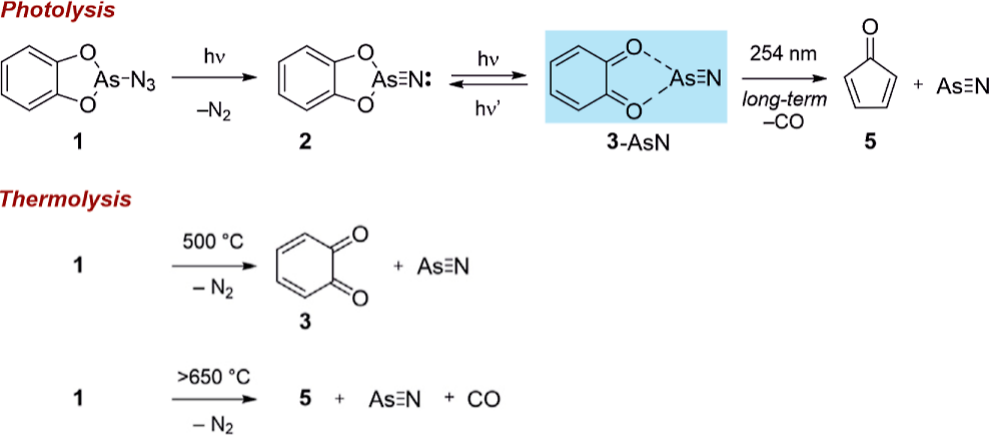
Photochemical and Thermal Generation of **2** and **3** from **1** and Subsequent Reactivity

## Results and Discussion

*ortho*-Phenyldioxoarsino azide (**1**) was synthesized (for details, see the Supporting Information) and isolated in an argon matrix at 10 K. The IR spectrum of matrix-isolated **1** is characterized by intense absorption bands centered at 2108.1 cm^–1^, in good agreement with the computed spectrum at the B3LYP/def2-TZVP level (Figures 2 and S1 and S2). When a matrix of **1** is exposed to light at a wavelength of 546 nm, new peaks at 1668.2/1666.4, 1411.8, 1276.5/1267.8, 1141.9, 1119.4, and 725.8 cm^–1^ emerge. By comparison with the computed spectrum at B3LYP/def2-TZVP, this new set of peaks was tentatively assigned to the *ortho*-benzoquinone-AsN complex (**3**-AsN, Figure 2). Considering the position of the C=O symmetric stretching band (1668.8 cm^–1^) in the previously reported *ortho*-benzoquinone–PN complex,^14^ the peak at 1668.2 cm^–1^ can be attributed to the analogous stretching vibration in **3**-AsN. We also identified a notably weak band at 1120.5 cm^–1^, corresponding to the As≡N stretching vibration mode in **3**-AsN. Note that in the ^15^N-isotope labeling experiments, this band exhibits a moderate red shift of 35.5 cm^–1^, which agrees well with the calculated isotopic shift of 33.6 cm^–1^ (Figure S3).

**Figure 2:**
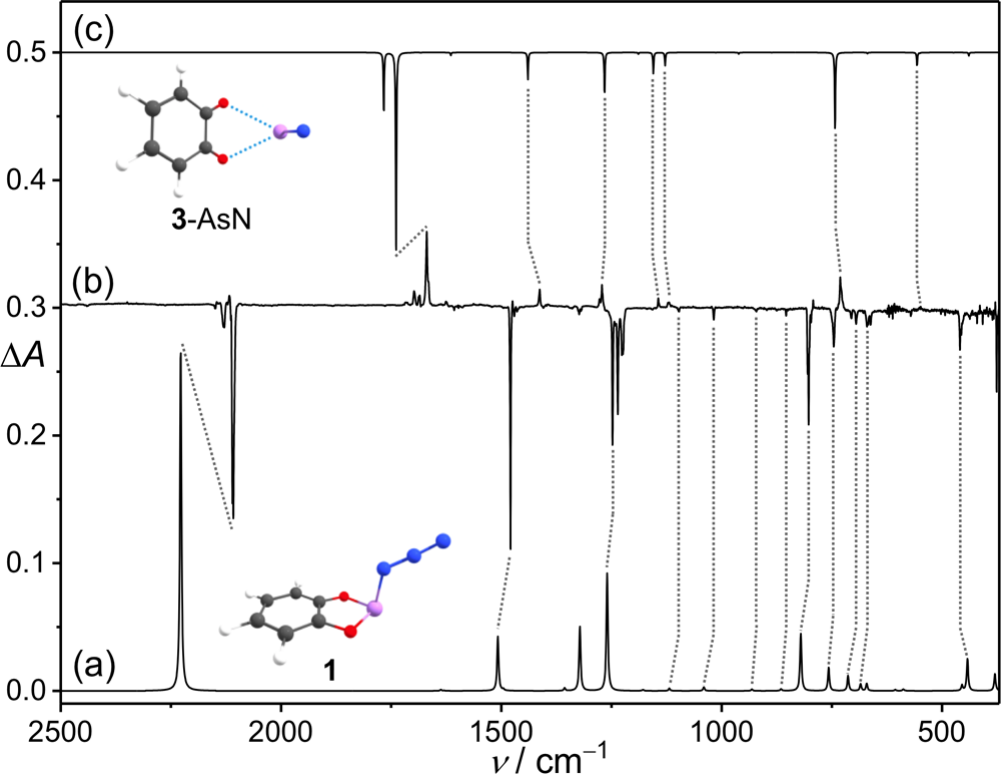
(a) Unscaled computed infrared spectrum for **1** at B3LYP/def2-TZVP. (b) Difference IR spectrum showing the changes after 15 min of 546 nm irradiation. (c) Unscaled computed infrared spectrum for **3**-AsN at B3LYP/def2-TZVP.

The **3**-AsN complex was also examined by UV/vis spectroscopy (Figure 3). Tallying with the infrared spectrum, irradiation at λ = 546 nm results in the rapid disappearance of the transitions at 278 and 217 nm of **1** and the appearance of transitions at 375 (weak), 271 (moderate), and 250 nm (moderate), which correlates well with the values of the electronic excitations of **3**-AsN at 406 (*f* = 0.0323), 276 (*f* = 0.0135), and 240 nm (*f* = 0.0613) computed at TD-B3LYP/def2-TZVP. The computed electronic excitation at 685 nm (*f* = 0.0015) is too weak to be detected in the experimental UV/vis spectrum, which corresponds to an n → π* transition (Figures 3 and S4).

**Figure 3:**
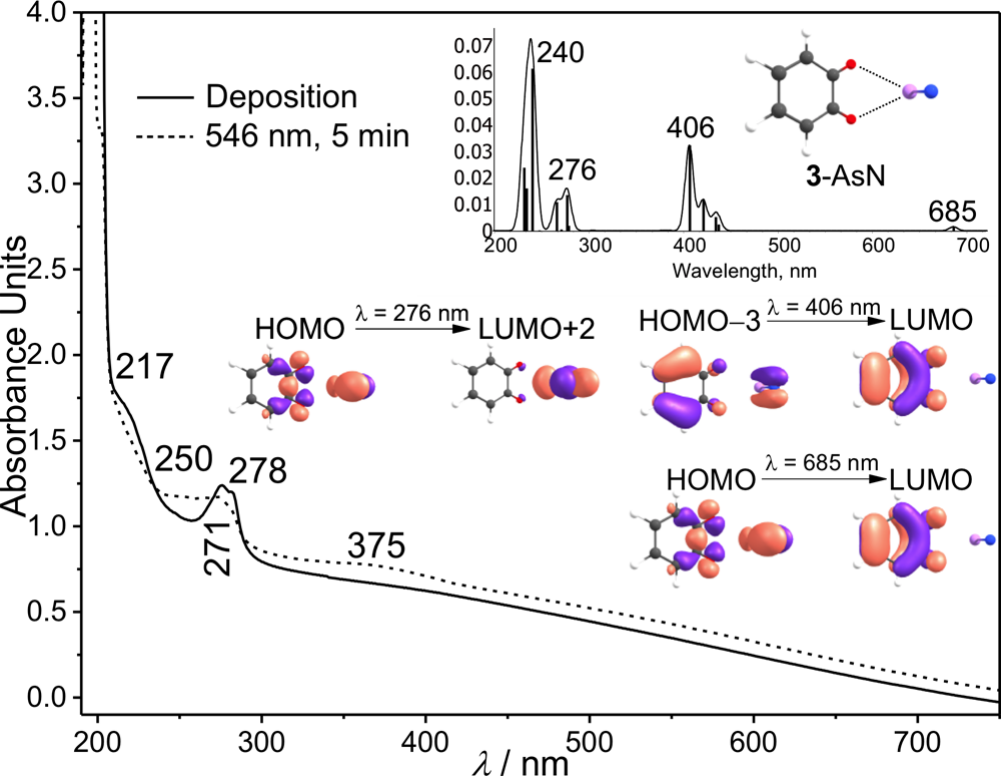
Solid line: UV/vis spectrum of **1** isolated in argon at 10 K. Dashed line: UV/vis spectrum of **3**-AsN at 10 K: photochemistry of **1** after irradiation at λ = 546 nm in argon at 10 K. Inset: Computed [TD-B3LYP/def2-TZVP] electronic transitions for **3**-AsN.

Upon photolysis of **1** by UV light (λ = 254 nm), ^1^ **2** and **3**-AsN form concomitantly (Figures S5 and S6). Though the IR bands for ^1^ **2** and **3**-AsN largely overlap with the strong bands of **1**, the strong absorptions at 1477.5, 743.9, and 673.9 cm^–1^ for ^1^ **2** and 1668.5 and 726 cm^–1^ for **3**-AsN are distinguishable. To gain more information about the photochemical properties of **3**-AsN, the mixture was subjected to photolysis with light of different wavelengths. In contrast to the previously reported **3**-PN complex, irradiation at λ = 546 nm did not cause recombination of **3**-AsN, but decomposition of ^1^ **2** (Figures S5 and S6).

Subsequent irradiation of **3**-AsN at λ = 654 nm shows the clean conversion of **3**-AsN to a new set of IR bands at 1477.5, 1318.7, 1235.6, 1100.0, 1023.8, 1018.9, 922.7, 849.6, 798.6, 743.9, 673.9, and 664.7 cm^–1^, which nicely matches the computed vibrations of singlet phenyldioxoarsino nitrene (**2**) at B3LYP/def2-TZVP (Figure S6). Computations at this level suggest that **2** has a Δ*E*_ST_ of 5.0 kcal mol^–1^, indicating a triplet electronic ground state. However, the new set of bands does not match the computed spectrum of ^3^ **2**. Notably, B3LYP, both with and without D3­(BJ)-corrections,^49^ consistently results in a triplet ground state (vide infra). Other levels of theory (B97-3c, M06-2X, PBE0, and ωB97M-V, all with a def2-TZVP basis set) show inconsistent results, giving either a closed-shell singlet or triplet ground state (Table S4).

To assess more accurately the energy gap between the singlet and triplet configurations of **2**, we conducted multireference computations based on the complete active space self-consistent field (CASSCF) and multistate complete active space second-order perturbation theory (CASPT2). We also used domain-based local pair-natural orbital singlets and doublet coupled clusters with a perturbative triplet correction (DLPNO-CCSD­(T)). The spin–orbit coupling (SOC) effects were included in order to correct the ground and excited state energies by allowing for the mixing of singlet and triplet states. These computations all suggest a closed-shell singlet ground state. At the multireference (MS)-CASPT2­(10,10)-SOC/ANO-S-VDZP level of theory, **2** exhibits a Δ*E*_ST_ of −6.8 kcal mol^–1^. The contribution of double (and higher multiple) excitations accounts for up to 13% (9%) of the singlet (triplet) ground-state configuration. Although these contributions are modest in magnitude, their combined effect, together with the multiconfigurational character of the states and the additional correlation captured at the PT2 level, leads to an inversion of the ground state relative to several of the tested DFT methods, which instead predict a triplet ground state (see Supporting Information). In the singlet configuration, the occupancy of the virtual orbitals in the active space is very small (up to ∼0.10); nevertheless, this configuration holds 0.71 unpaired electrons in total, manifesting the multiconfigurational character of the ground state of **2**, mixing open and closed-shell configurations (as the double excitations) of this state. DLPNO-CCSD­(T)/cc-pVTZ results also suggest the stabilization of the closed-shell singlet (Δ*E*_ST_ = −4.0 kcal mol^–1^), which is in very good agreement with the CASPT2 computations discussed above. Hence, DFT fails to properly describe the electronic structure of this singlet state. The second possible candidate that could be generated from the irradiation of **3**-AsN (λ = 546 nm) is benzo­[1,4,2,3]­dioxazarsinine (**4**). Interestingly, the computed spectrum for **4** is similar to that of ^1^ **2** (Figures 4 and S7). To exclude the possibility of misassignment of the photolysis results, we conducted experiments using (terminal *N*, 98 atom %) ^15^N_1_-labeled **1**. According to B3LYP/def2-TZVP computations, the N–O stretching mode in **4** (1030.5 cm^–1^) and the As≡N stretching mode in ^1^ **2** (1047.4 cm^–1^) are close. The ^15^N-isotope labeling predominantly affects the As≡N stretching mode, while other modes are largely unaffected. For example, the ring distortion mode at 1041.4 cm^–1^ (0.1 cm^–1^ red-shifted), deformation mode of the O–As–O moiety at 808.0 cm^–1^ (0.1 cm^–1^ blue-shifted), and the symmetric stretching mode of the O–As–O moiety at 684.4 cm^–1^ (0.5 cm^–1^ blue-shifted) fit nicely with the computed shifts. The other computed vibrations display no shifts (Figure 4 and Table S2). In contrast, the nitrogen atom in **4** is directly bonded to the arsenic and oxygen atoms in a ring system, leading to larger observed isotopic shifts. In particular, the calculated isotopic shift of the NO stretching mode in **4** is 18.7 cm^–1^, whereas that of the AsN stretching mode in ^1^ **2** is 28.6 cm^–1^. The experimentally observed shift for the band at 1023.8 cm^–1^ is 30.9 cm^–1^, which better matches the calculated shift of 28.6 cm^–1^ for ^1^ **2** (Figure 4 and Table S3). The peak shifts at 1018.9 (0.1 cm^–1^), 798.6 (0.1 cm^–1^), and 673.9 cm^–1^ (0.5 cm^–1^) also agree well with the computed values of −0.1, 0.1, and 0.5 cm^–1^ for ^1^ **2**, respectively. Based on these analyses, we conclude that the new set of IR bands appearing upon irradiation of **3**-AsN at λ = 654 nm can be assigned to ^1^ **2** rather than to **4**.

**Figure 4:**
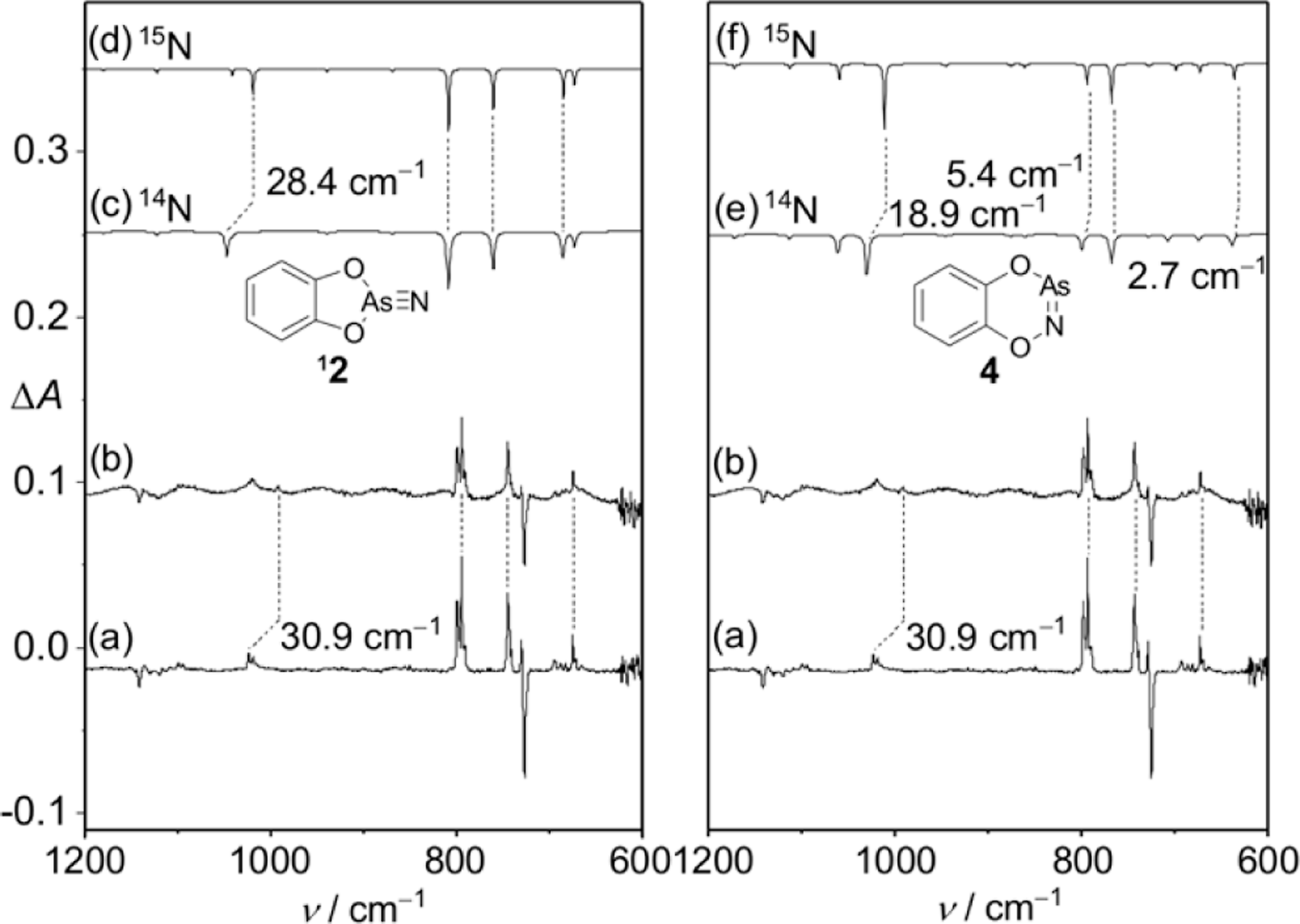
(a) Difference IR spectrum showing the changes after 10 min of 654 nm irradiation. (b) Difference IR spectrum showing the changes after 10 min of 654 nm irradiation for the ^15^N-labeling sample. (c) Unscaled computed infrared spectrum of ^1^ **2**. (d) Unscaled computed infrared spectrum for ^15^N-^1^ **2**. (e) Unscaled computed infrared spectrum for **4**. (f) Unscaled computed infrared spectrum for ^15^N-**4**.

To better understand the chemistry involved, we computed the potential energy surface around **2** at the B3LYP-D3/def2-TZVP level of theory (Figure 5). Based on these computations, **1** can exist in *anti*-(**1a**) and *syn* (**1b**) conformations, differing in the orientation of the azide group relative to the opposing phenyl group; **1a** and **1b** display an energy difference of only 0.9 kcal mol^–1^ in favor of **1b**. The rotational barrier connecting these two conformations is less than 2 kcal mol^–1^ (Δ*H*_0_^‡^). The first reaction path is the cleavage of N_2_ from **1b**, leading to the formation of closed-shell singlet nitrene ^1^ **2** through transition state **TS2** with a barrier of 46.3 kcal mol^–1^. The subsequent dissociation of AsN results in the formation of the **3**-AsN complex that is associated with a dissociation energy (*D*_0_) of 3.4 kcal mol^–1^; the barrier of this process amounts to 9.2 kcal mol^–1^. Furthermore, a two-dimensional relaxed scan along the O1–As and O2–As bonds in ^1^ **2** at RI-B3LYP/def2-TZVP revealed a saddle point along the concerted path, indicating that the decomposition of ^1^ **2** into **3**-AsN is indeed a concerted process (Figure S8). The formation of **4** from ^1^ **2** is a one-step process with a barrier (**TS4**) of 17.3 kcal mol^–1^. Compound **4** can also form either directly from **1a**, with the reaction requiring an activation energy of 50.1 kcal mol^–1^ (**TS5**) or from the combination of free AsN with **3** by overcoming a barrier of only 9.6 kcal mol^–1^ (**TS6**). The pyrolysis temperature of 850 °C facilitates the additional decarbonylation of **3**, resulting in infrared signals attributed to the formation of cyclopentane-2,4-dienone (**5**). The dissociation is characterized by a high-lying transition state (Δ*H*_0_^‡^ = 61.6 kcal mol^–1^, **TS7**, Scheme 1 and Figure S9).

**Figure 5:**
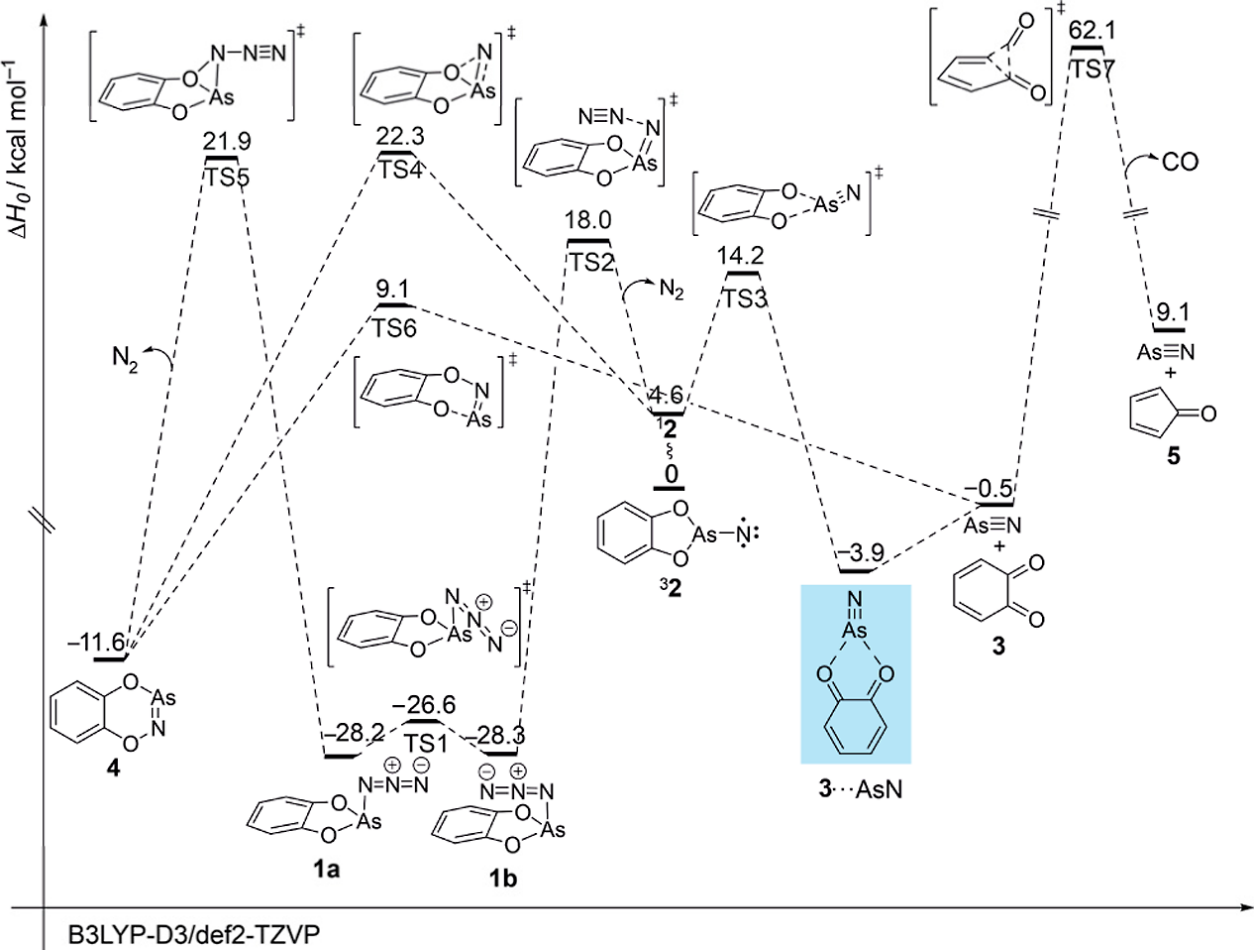
Potential energy hypersurface profile (Δ*H*_0_, kcal mol^–1^) of the reactions of **2** at B3LYP-D3/def2-TZVP + ZPVE.

We have also conducted a comparative analysis of the essential structural parameters of ^1^ **2** and ^3^ **2** with the recently acquired phosphinonitrene (**6**) (Figure 7).^14^ Arsenic forms weaker and longer bonds than phosphorus with nitrogen, owing to the larger atomic radius of arsenic with larger and more diffuse orbitals, resulting in poorer orbital overlap with the nitrogen orbitals compared to the phosphorus atom. The AsN bond distances in ^1^ **2** and ^3^ **2** are 1.625 and 1.705 Å, respectively, at B3LYP/def2-TZVP, thus showing double-bond character in both cases (Mayer bond indices of 2.5 and 1.9).^50^ This differs markedly from the previously reported nitrene **6**,^14^ where the PN bond length in singlet ^1^ **6** (1.483 Å) is notably shorter than in triplet ^3^ **6** (1.665 Å). The corresponding Mayer bond indices for ^1^ **6** and ^3^ **6** are 2.47 and 1.61, respectively, indicating quite different bond characters between singlet and triplet states. The AsN bond distance in ^1^ **2** is comparable to the corresponding values in previously reported compounds with an anionic arsa-diazonium salt (**B**) of 1.613 Å.^46^ The differences between the geometries of ^1^ **2** and ^3^ **2** are much smaller than those in ^1^ **6** and ^3^ **6**, reflecting the weaker π-bonds in the arsenic congener as compared to phosphorus. Indeed, the HOMO (highest occupied molecular orbitals) in ^3^ **2** is an in-plane orbital combining the σ­(As–N) orbital, the As lone pair, and the in-plane π-orbital of the phenyl ring. The HOMO in ^1^ **2** is above the phenyl ring and displays π-bonding between the arsenic and nitrogen atoms. When phosphorus is present instead of arsenic, the HOMO atom in ^1^ **6** displays pronounced π-bonding between the phosphorus and nitrogen atoms. Overall, the MOs of **2** show clear evidence for the tendency of the heavier elements to avoid multiple bonding (Figures 6 and S10).

**Figure 6:**
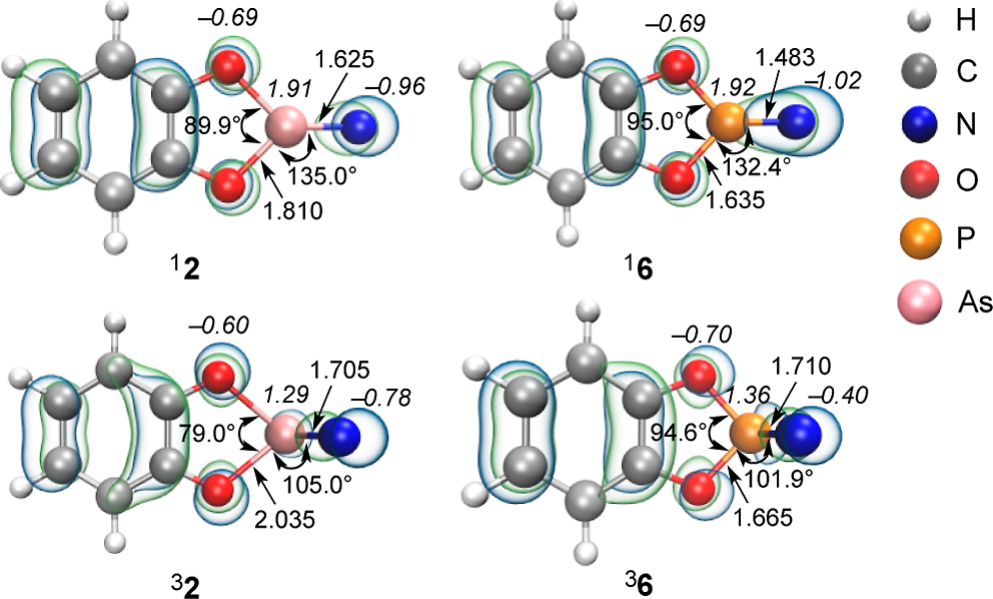
HOMO (alpha-HOMO for triplet species), selected bond lengths [Å], and angles of **2** and **6** at B3LYP/def2-TZVP. Atomic charges computed through the natural population analysis (NPA) are in italics.

**Figure 7:**
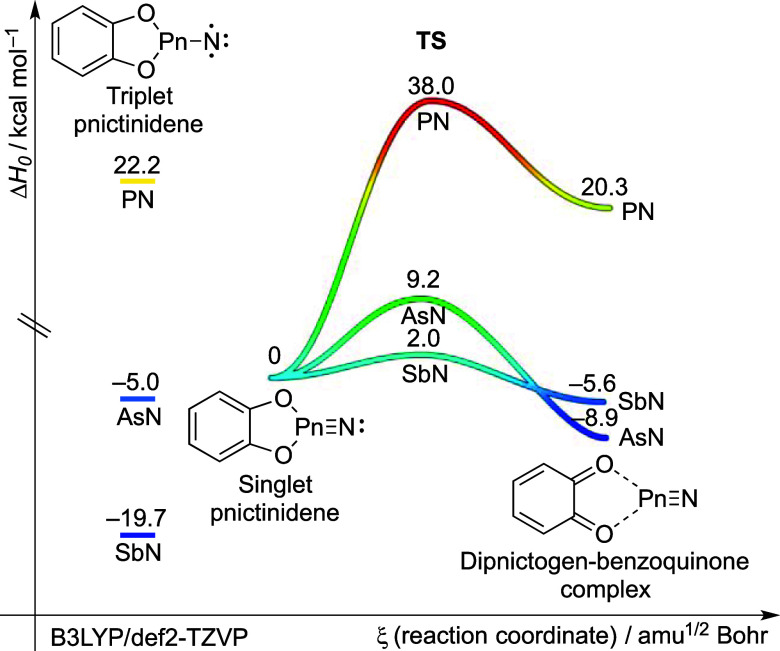
Potential energy hypersurface profile (Δ*H*_0_, kcal mol^–1^) of the reactions of dipnictogens at B3LYP/def2-TZVP + ZPVE.

## Conclusions

We report the preparation of AsN weakly complexed to *ortho*-benzoquinone (**3**-AsN) upon 254/546 nm irradiation of *ortho*-phenyldioxoarsinoazide (**1**), which subsequently undergoes recombination to yield new *ortho*-phenyldioxoarsino nitrene (**2**) by 654 nm excitation, which displays a closed-shell singlet state. The formation of ^1^ **2** is also supported by isotopic labeling experiments using ^15^N-**1**. To determine the ground state of **2**, singlet–triplet energy gaps were evaluated by using both single-reference and multiconfigurational methods. Higher-level computations incorporating SOC corrections indicate a singlet ground state, with energy singlet–triplet gaps of 6.8 and 4.0 kcal mol^–1^ obtained at the MS-CASPT2­(10,10)-SOC/ANO-S-VDZP and DLPNO-CCSD­(T)/cc-pVTZ levels of theory, respectively.

Our method is generally applicable for the synthesis and preparation of heavy dipnictogens such as antimony nitride (SbN) (Figures 7 and S11). Computational results suggest that the formation of heavier dipnictogens from the corresponding singlet pnictinidenes (nitrenes, phosphinidenes, stibinidenes) is characterized by higher exothermicity and lower activation barriers. Therefore, this approach opens avenues for the straightforward synthesis of various dichalcogenides and thus the development of new pnictogen materials. Further studies on the synthesis and characterization of higher dipnictogens are currently underway.

## Supplementary Material



## Notes

Further experimental, spectroscopic, and computational data (PDF)
