# Supplementary material for: Synthesis of an o‑Benzoquinone Arsenic Mononitride (As≡N) Complex and Its Reaction to Singlet Arsinonitrene
Source: J Am Chem Soc. 2026 Mar 10;148(11):11719–25. doi: 10.1021/jacs.5c20377 (PMC13022887; doi:10.1021/jacs.5c20377)
Supplement: Supplementary file 1 [file ja5c20377_si_001.pdf]

Supporting Information

Synthesis of an o-Benzoquinone Arsenic Mononitride (As≡N) Complex and Its Reaction to Singlet Arsinonitrene

Weiyu Qian,[a] Maria Eugenia Sandoval-Salinas,[b] Rachel Crespo-Otero,[b] Peter R. Schreiner,[a] and Artur Mardyukov[a]*

[a]Institute for Organic Chemistry, Justus Liebig University, Heinrich-Buff-Ring 17, 35392 Giessen (Germany)

[b]Department of Chemistry, University College London, London WC1H 0AJ (United Kingdom)

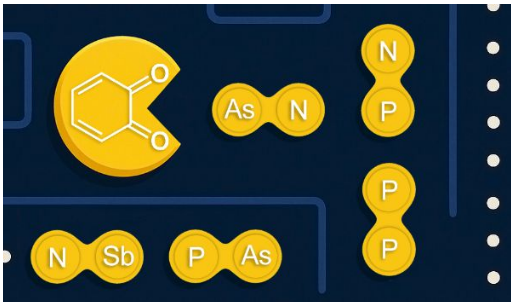

Experimental Section

Matrix Apparatus Design. For the matrix isolation studies we used an APD Cryogenics HC-2 cryostat with a closed-cycle refrigerator system, equipped with an inner CsI window for IR measurements. Spectra were recorded with a Bruker Vertex 70 FT-IR spectrometer with a spectral range of 4000−400 cm^{-1} and a resolution of 0.7 cm^{-1} and UV/Vis spectra were recorded with a JASCO V-670 spectrophotometer equipped with an inner sapphire window. A high-pressure mercury lamp (HBO 200, Osram) with a monochromator (Bausch & Lomb) was used for irradiation with a nominal wattage of 200 W. For the combination of high-vacuum flash pyrolysis with matrix isolation, we employed a small, homebuilt, water-cooled oven, which was directly connected to the vacuum shroud of the cryostat. The pyrolysis zone consisted of an empty quartz tube with an inner diameter of 8 mm, which was resistively heated over a length of 50 mm by a coaxial wire. The temperature was monitored with a NiCr−Ni thermocouple. Azides were evaporated (1: 0 °C) from a storage bulb into the quartz pyrolysis tube. At a distance of approximately 50 mm, all pyrolysis products were co-condensed with a large excess of argon (typically 60−120 mbar from a 2000 mL storage bulb) on the surface of the matrix window at 10 K.

Caution! *Covalent azides are extremely hazardous explosives. Although we have not experienced any incident during this work, they should be handled with great care in small quantities (< 5 mmol) and safety precautions (face shields, Kevlar gloves, and protective leather clothing) are strongly recommended. Arsenic compounds are extremely toxic and environmentally hazardous that should be handled in a well-vented fume hood with great care and all waste must be collected and disposed appropriately.*

Synthesis of (o-phenyldioxyl)arsinoazide 1. In a flame-dried Schlenk flask, 0.130 g (2 mmol) NaN_3 and 0.218 g (1 mmol) sublimed *o*-phenylene chloroarsenate were suspended in 2.00 mL anhydrous CH_3CN and stirred for 24 h. Filtration through a syringe filter afforded a colorless solution. This was cooled to 0 °C and brought to reduced pressure to remove solvent affording a pale gray solid. This synthesis was repeated several times and essentially provided quantitative

yields. The purity of the obtained product was confirmed by NMR (^{1}H NMR (600 MHz, CD_3CN): δ = 6.83 (m, 2 H), 6.70 (m, 2 H). ^{13}C NMR (151 MHz, CD_3CN): δ = 145.84, 121.13, 116.86 ppm).

Computations. A 2D relaxed energy scan of **2** was performed using ORCA 5.01 employing the B3LYP/G keywords combined with the Resolution of Identity (RI) approximation (RIJCOSX). Other B3LYP2 computations were carried out with Gaussian16 [3] with a def2-TZVP basis set.[4] Local minima were confirmed by vibrational frequencies analyses, and transition states were further confirmed by intrinsic reaction coordinate (IRC) calculations. A dispersion correction was applied via the D3-BJ Grimme scheme.[5] Wavefunction analyses were done using Multiwfn 3.8.6

Adiabatic S-T energy gap (ΔE_{S-T}) were determined with the B97-3c[7], M06-2X[8], PBE0,[9] ωB97M-V[10] and B3LYP functionals the def2-TZVP basis set including dispersion correction trough the D3BJ scheme.[11] The computations using the CASSCF and CASPT2 methods with SOC corrections were carried out with active spaces including 10(12) electrons in 10(12) orbitals.[12] The multistate approach was employed considering the lowest two singlet and triplet electronic states; these computations were done with the ANO-S-VDZP basis set using the Open Molcas version 23.02 software.[12] DFT and DLPNO-CCSD(T)/cc-pVTZ[13] computations were done with the def2-TZVP basis set and corrected by including the SOC contributions with the ORCA 5.0 program.

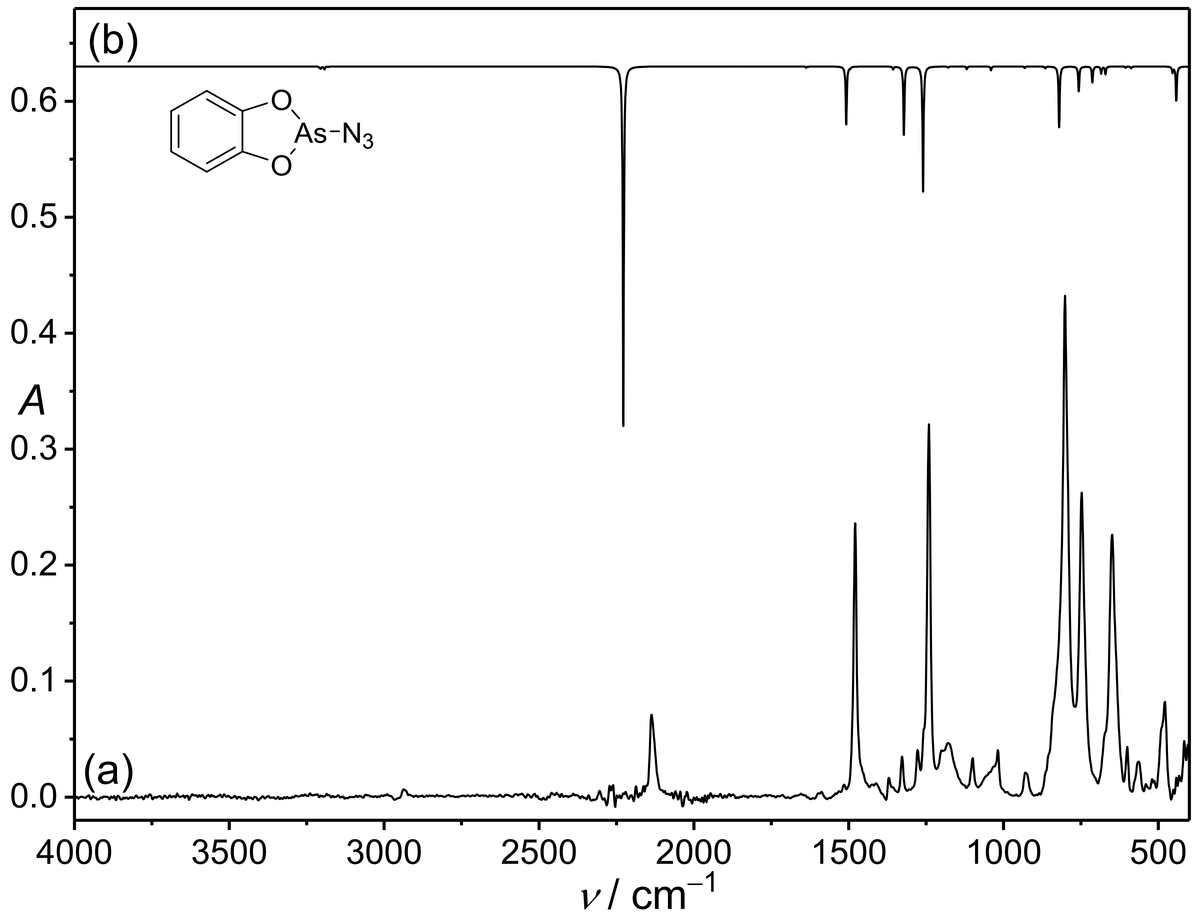

Figure S1. (a) ATR-IR spectrum for (o-phenyldioxyl)arsinoazide (**1**) in CH_3CN (solvent spectrum subtracted). (b) Unscaled computed infrared spectrum for **1**.

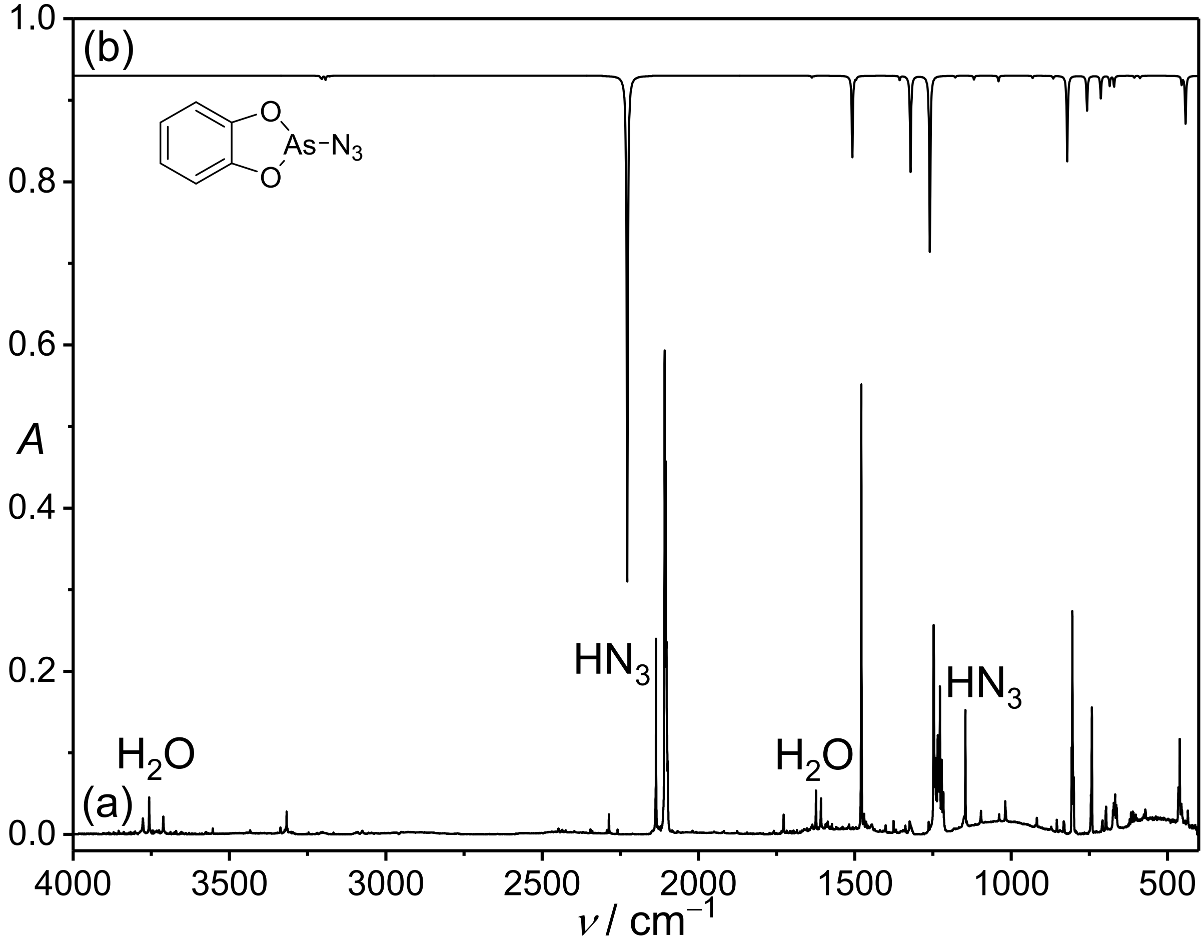

Figure S2. (a) IR spectrum for (o-phenyldioxyl)arsino azide (**1**) in the Ar-matrix. (b) Unscaled computed infrared spectrum for **1**.

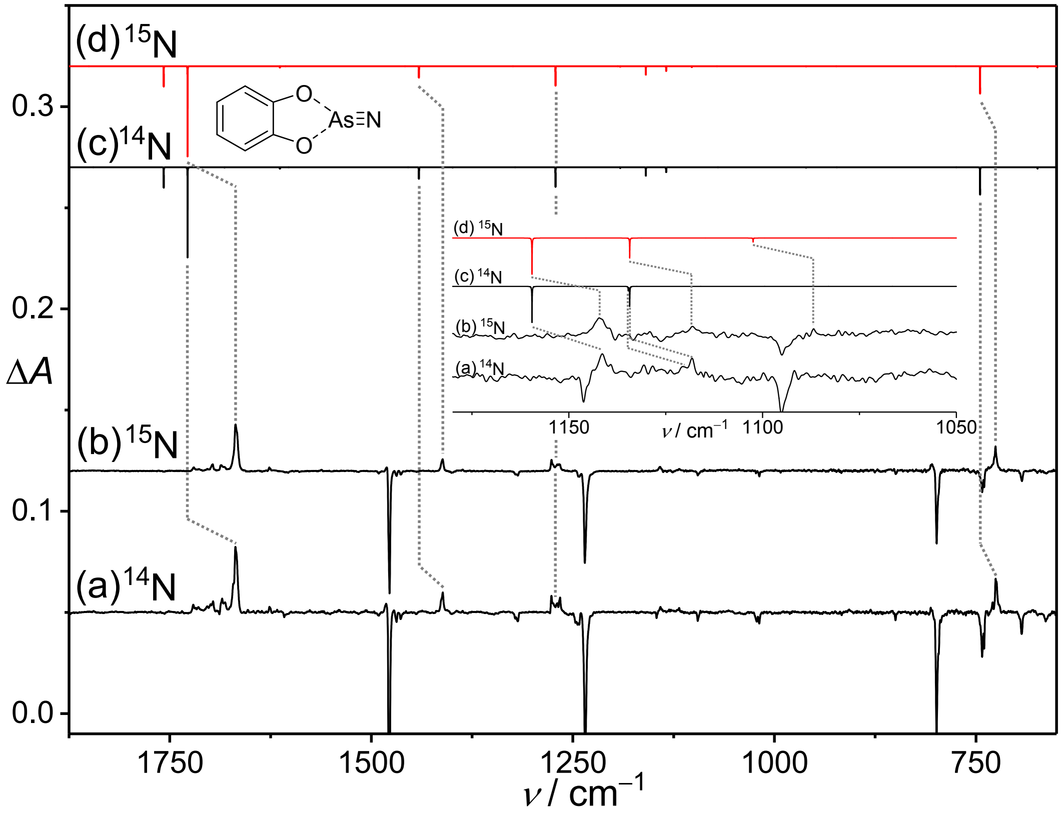

Figure S3. (a) Difference IR spectrum shows the changes of subsequent 4 min of 546 nm irradiation after 10 min 254 nm irradiation of **1**. (b) Difference IR spectrum shows the changes of subsequent 4 min of 546 nm irradiation after 10 min of 254 nm irradiation of a ^{15}N-labeled **1**. (c) Unscaled computed infrared spectrum of **3**-AsN in natural abundance. (d) Unscaled computed infrared spectrum for ^{15}N-labeled **3**-AsN. Inset: expanded spectra multiplied by nine.

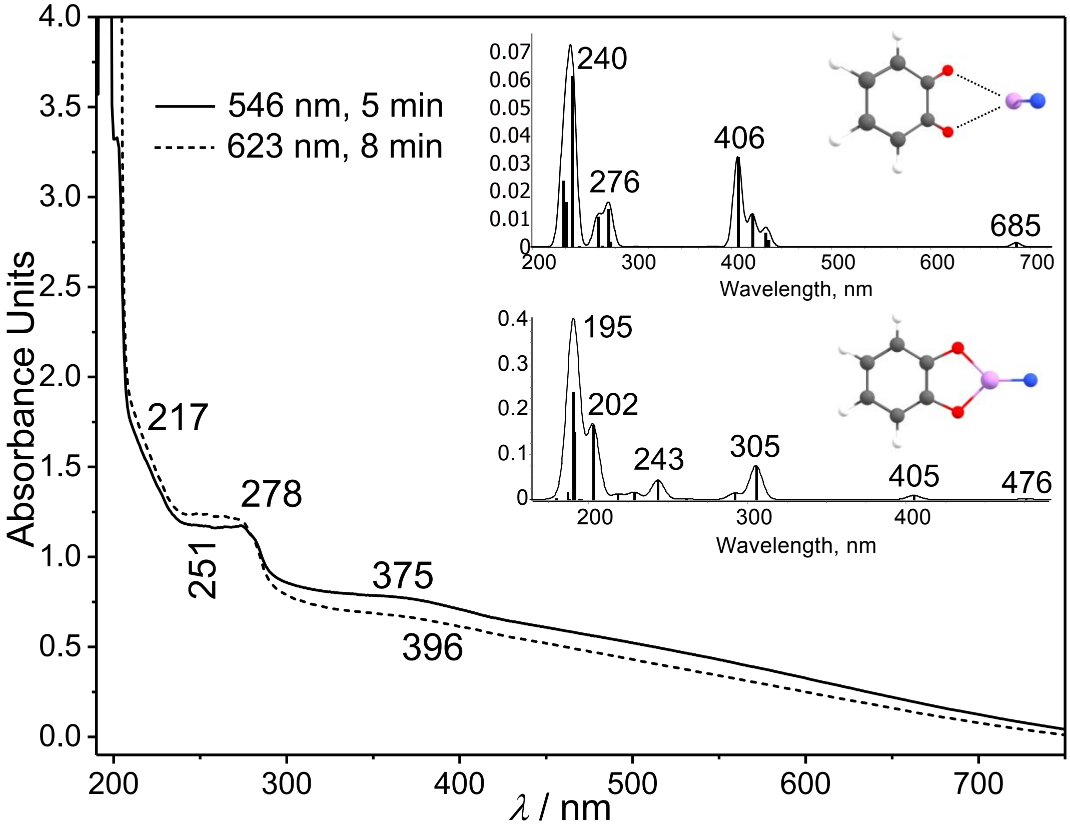

Figure S4. Solid line: UV/Vis spectrum of **2** isolated in an Ar-matrix. Dashed line: UV/Vis spectrum for **3-**AsN isolated in an Ar-matrix. Inset: Computed [TD-B3LYP/def2-TZVP] electronic transitions.

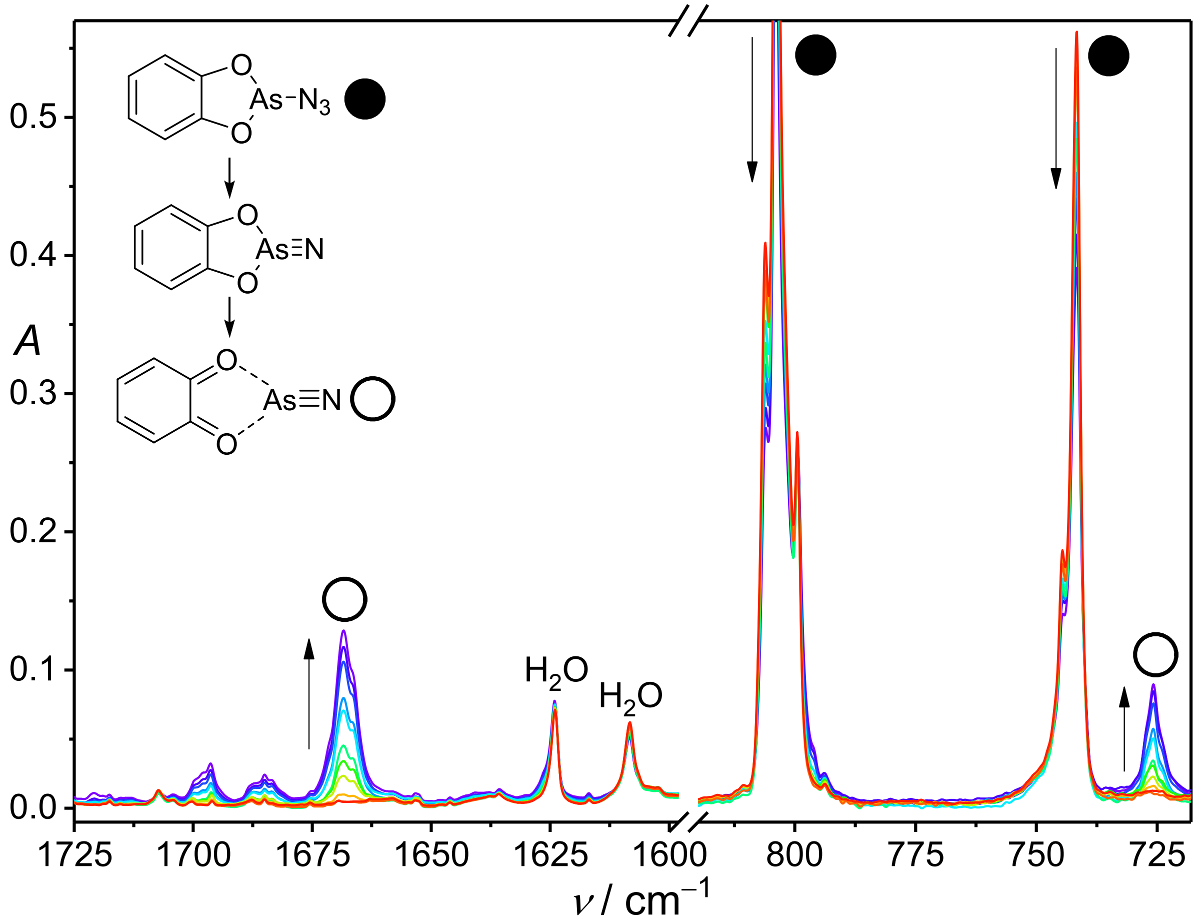

Figure S5. Time-dependent infrared spectra upon 254 nm irradiation of **1** in an Ar-matrix (10 K).

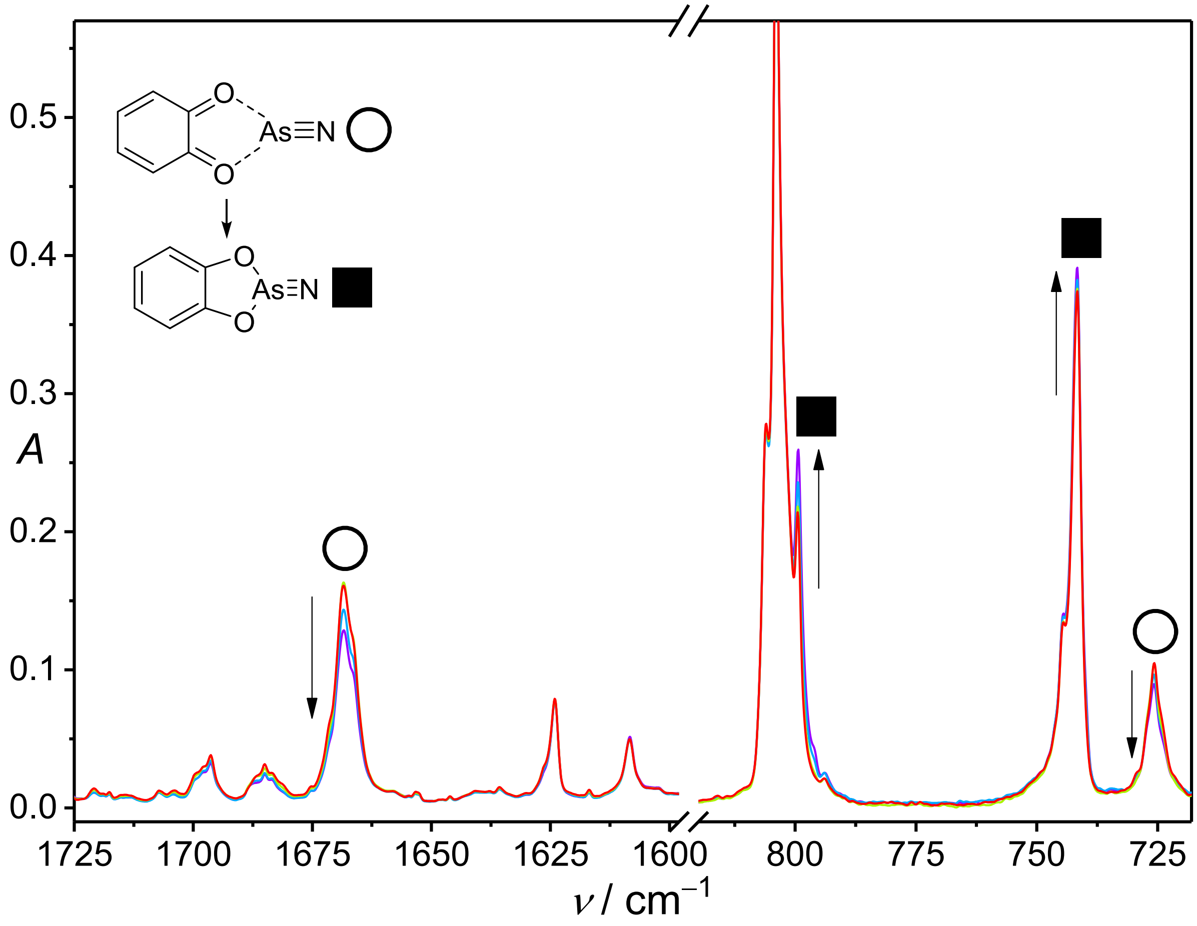

Figure S6. Time-dependent infrared spectra upon subsequently 648 nm irradiation after 254 nm irradiation of **1** in an Ar matrix (10 K).

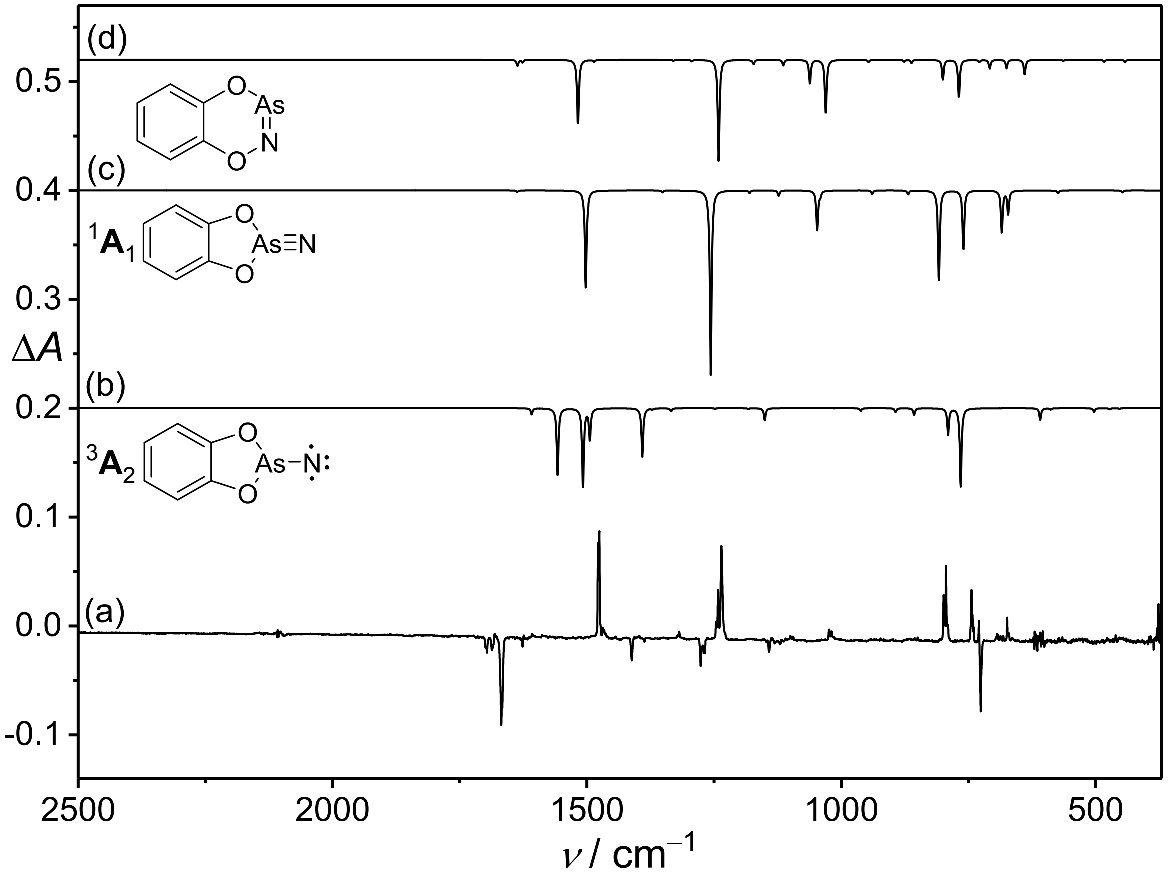

Figure S7. (a) Difference IR spectrum showing the changes after 10 min of 654 nm irradiation. (b) Unscaled computed infrared spectrum of $^{3}A_{2}$-**1**. (c) Unscaled computed infrared spectrum of $^{1}A_{1}$-**1**. (d) Unscaled computed infrared spectrum for **4**.

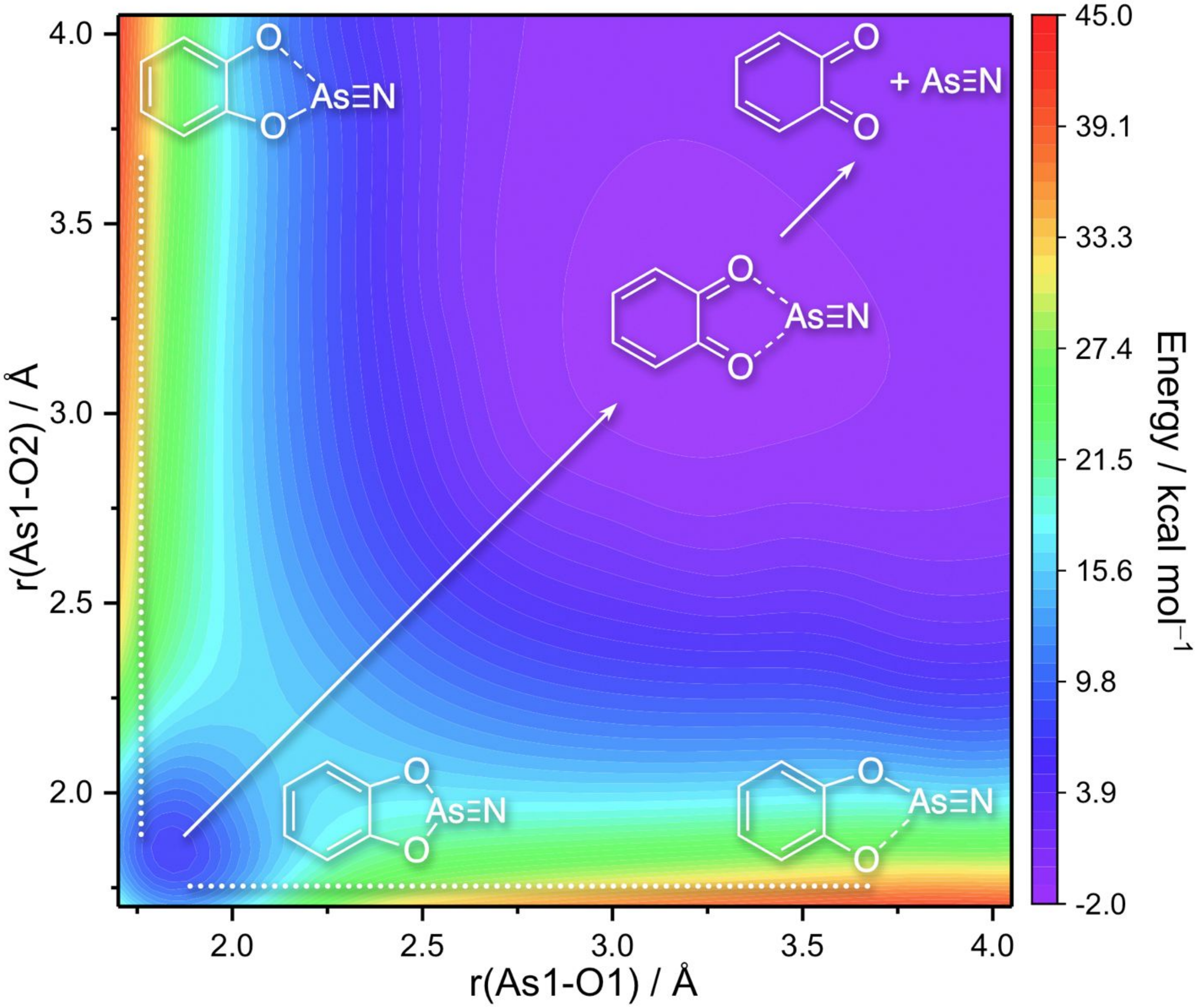

Figure S8. 2D relaxed energy scan of the As1-O1 and As1-O2 bonds at RI-B3LYP/def2-TZVP .

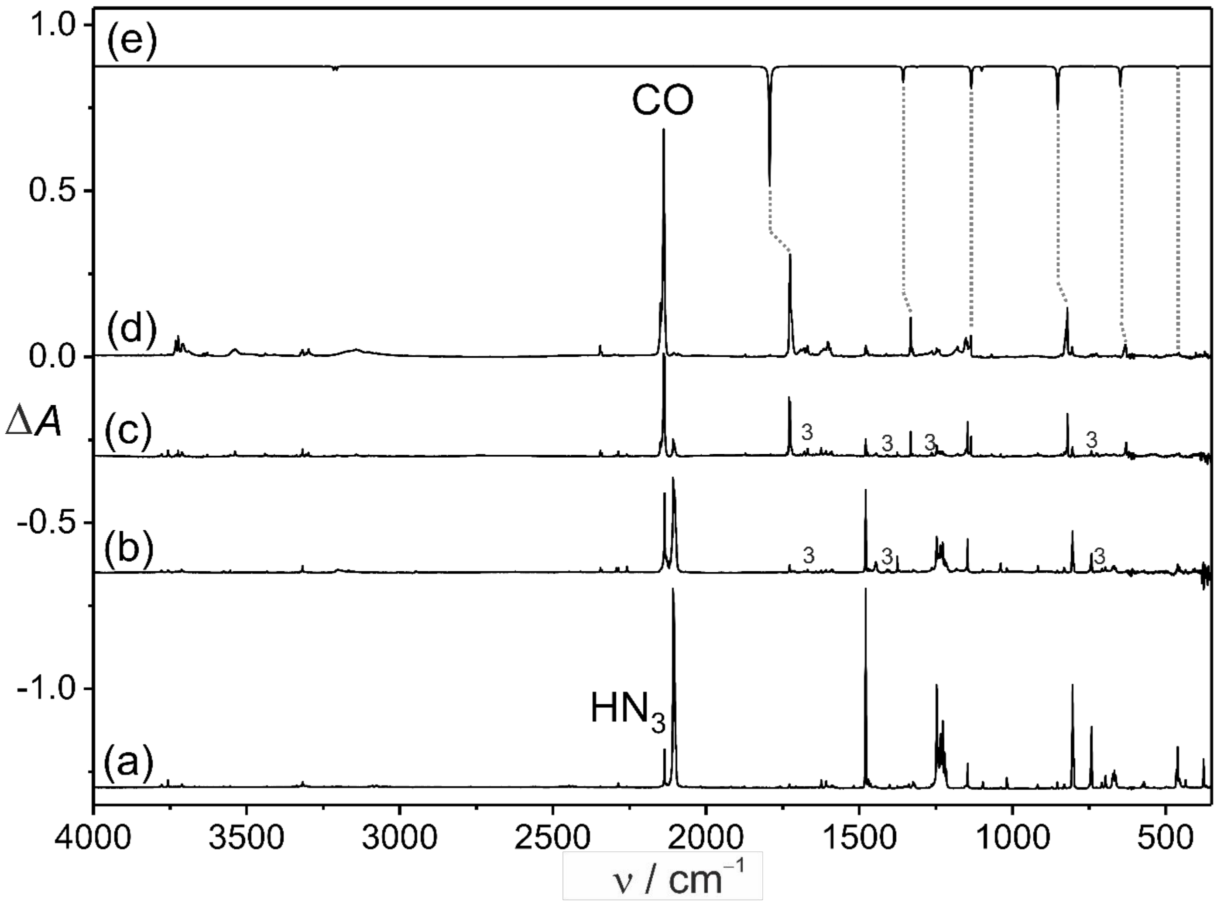

Figure S9. (a) IR spectrum of **1** in an Ar-matrix. (b) IR spectrum of HVFP (450 °C) product of **1** in an Ar-matrix. (c) IR spectrum of HVFP (650 °C) product of **1** in an Ar-matrix. (d) IR spectrum of HVFP (850 °C) product of **1** in an Ar-matrix. (e) Unscaled computed infrared spectrum of cyclopentadienone.

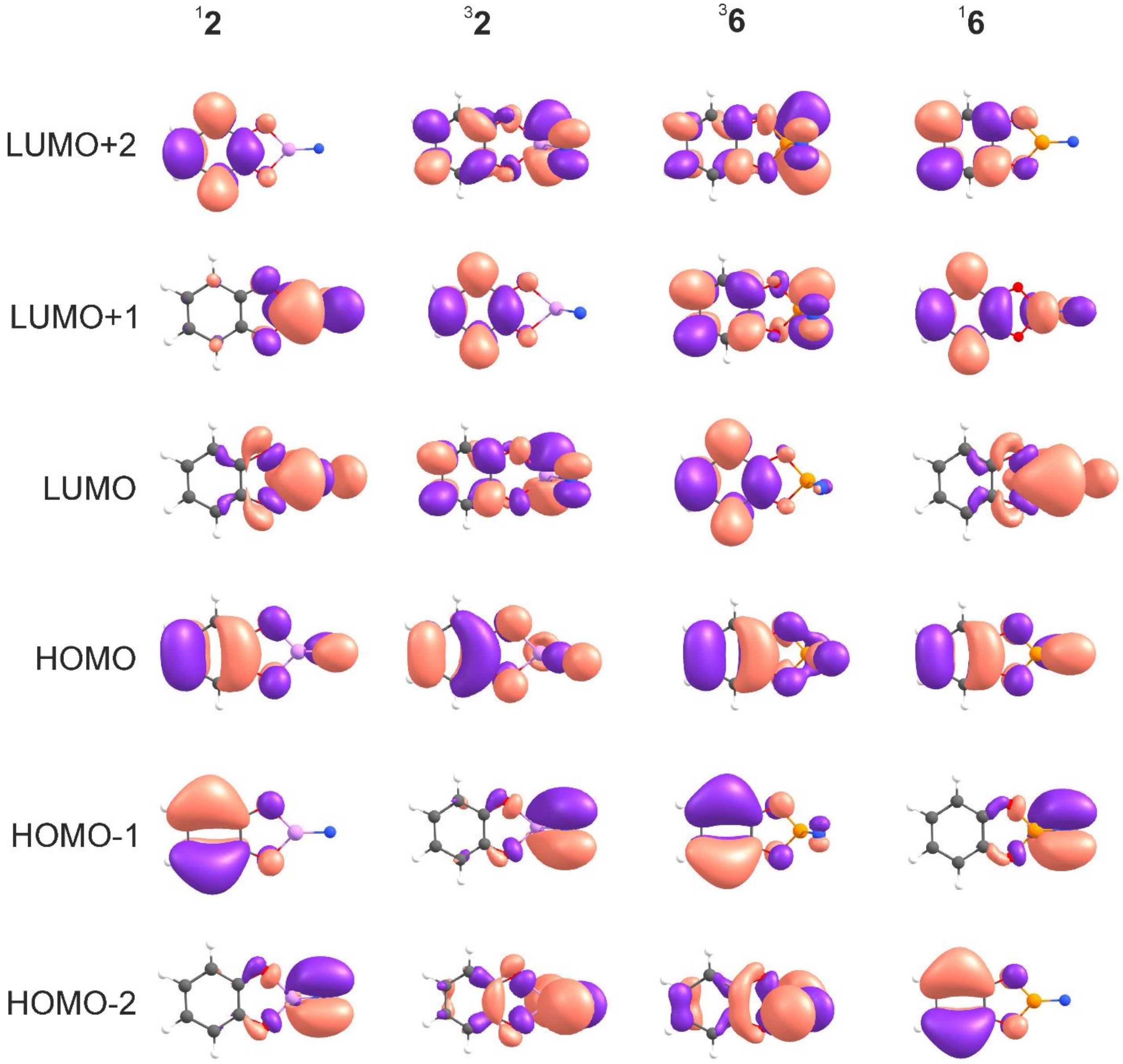

Figure S10. Computed molecular orbitals for ^{1}**2** , ^{3}**2**,^{1}**6** , ^{3}**6** at the B3LYP/def2-TZVP level of theory.

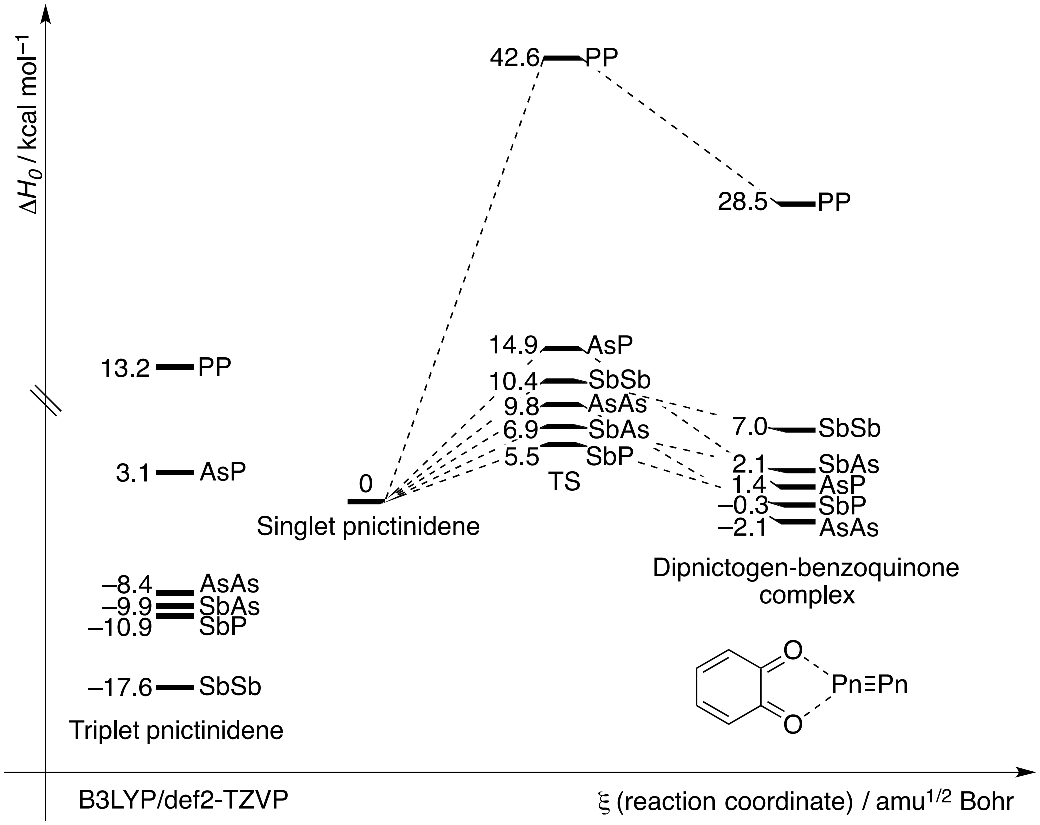

Figure S11. Potential energy hypersurface profile of the reactions of dipnictogens with ortho-benzoquinone at B3LYP/def2-TZVP + ZPVE.

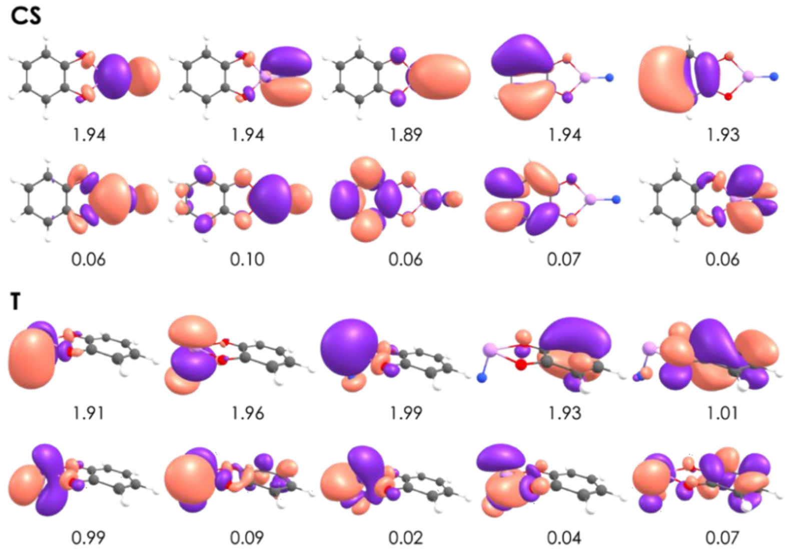

Figure S12. Natural orbitals of **2** with occupancy numbers included in the active space (10 electrons in 10 orbitals) of MS-CASPT2/ANO-S-VDZP computations.

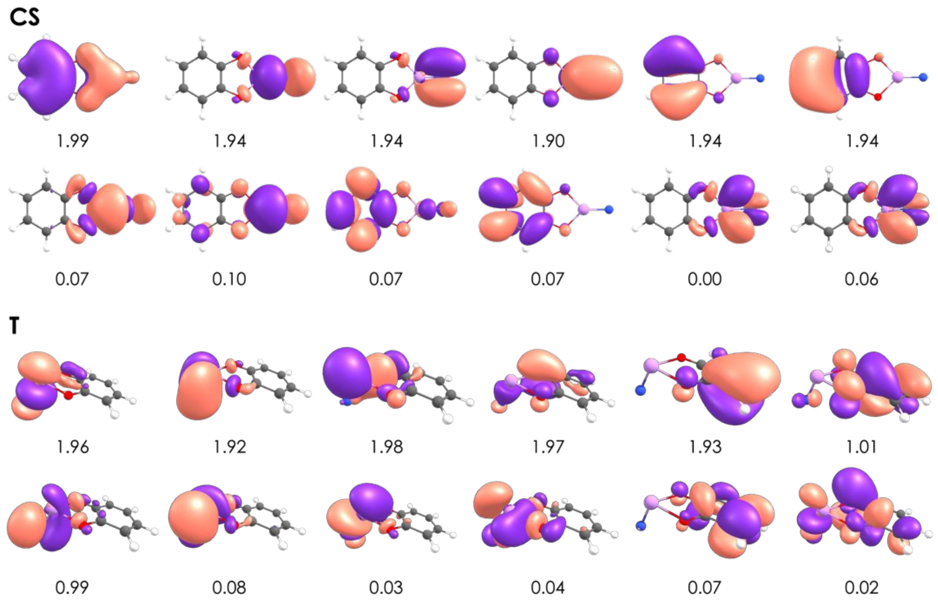

Figure S13. Natural orbitals of **2** with occupancy numbers included in the active space (12 electrons in 12 orbitals) of the MS-CASPT2/ANO-S-VDZP computations.

Table S1. Experimental and Computed IR frequencies (> 400 cm^{-1}) and intensities (km mol^{-1}) for singlet ortho-phenyldioxylarsenonitrene (^{1}**2**).

Mode	$\nu_{theor.}$<br>B3LYP	$\nu_{exp.}$<br>Ar-matrix	$\Delta\nu_{theor.}$<br>$^{15}N/^{14}N$<br>B3LYP	$\Delta\nu_{exp.}$<br>$^{15}N/^{14}N$<br>Ar-matrix	assignment
36	3211.9 (3)	n.o.	0.0	n.o.	a1, CH str.
35	3208.5 (2)	n.o.	0.0	n.o.	b2, CH str.
34	3196.8 (7)	n.o.	0.0	n.o.	a1, CH str.
33	3184.0 (1)	n.o.	0.0	n.o.	b2, CH str.
32	1637.1 (2)	n.o.	0.0	n.o.	a1, C=C str.
31	1633.1 (< 1)	n.o.	0.0	n.o.	b2, C=C str.
30	1502.3 (129)	1477.5 (vs)	0.0	0.0	a1, C=C str. / CH def.
29	1498.1 (5)	n.o.	0.0	n.o.	b2, C=C str. / CH def.
28	1351.7 (3)	1318.7 (w)	0.0	0.0	a1, C=C str.
27	1305.7 (< 1)	n.o.	0.0	n.o.	b2, CH def.
26	1256.5 (246)	1235.6 (vs)	0.0	0.0	a1, CO def.
25	1187.9 (< 1)	n.o.	0.0	n.o.	b2, CH def.
24	1180.4 (3)	n.o.	0.0	n.o.	a1, CH def.
23	1123.0 (7)	1100.0 (w)	0.0	0.0	b2, CH def.
22	1047.4 (53)	1023.8 (m)	28.6	30.9	a1, AsN str.
21	1041.4 (6)	1018.9 (w)	−0.1	0.1	a1, ring distortion
20	957.0 (< 1)	n.o.	0.0	n.o.	a2, CH o.o.p. def.
19	939.1 (5)	922.7 (w)	0.0	0.0	b1, CH o.o.p. def.
18	869.8 (< 1)	n.o.	0.0	n.o.	a2, CH o.o.p. def.
17	868.5 (5)	849.6 (w)	0.0	0.0	b2, ring distortion
16	808.0 (121)	798.6 (vs)	0.1	0.1	a1, OAsO def.
15	759.8 (80)	743.9 (s)	0.0	0.0	b1, CH o.o.p. def.
14	742.8 (< 1)	n.o.	0.0	n.o.	a2, CH o.o.p. def.
13	684.4 (56)	673.9 (s)	0.5	0.5	a1, OAsO sym. str.

12	671.9 (31)	664.7 (w)	0.0	0.0	b2, OAsO assym. str.
11	584.8 (1)	n.o.	0.0	n.o.	a1, ring distortion
10	573.8 (4)	n.o.	0.0	n.o.	b2, ring distortion
9	572.8 (< 1)	n.o.	0.0	n.o.	a2, ring breathing
8	447.4 (3)	n.o.	0.0	n.o.	b1, ring breathing

Table S2. Computed IR frequencies (> 400 cm^{-1}) and intensities (km mol^{-1}) for 1,4,3,2-benzodioxarsirine (**4**).

Mode	$\nu_{cal.}$ B3LYP	$\Delta\nu_{theor.}(^{15}N/^{14}N)$ B3LYP	assignment
36	3205.0 (8)	0.0	CH str.
35	3197.4 (5)	0.0	CH str.
34	3189.8 (5)	0.0	CH str.
33	3179.8 (1)	0.0	CH str.
32	1636.5 (12)	0.0	C=C str.
31	1626.5 (5)	0.0	C=C str.
30	1517.5 (133)	0.0	C=C str. / CH def.
29	1485.9 (4)	0.0	C=C str. / CH def.
28	1330.0 (2)	0.0	C=C str.
27	1293.8 (3)	0.0	CH def.
26	1241.0 (202)	0.1	CH def. / CO str.
25	1178.6 (1)	0.0	CH def.
24	1172.3 (9)	0.1	CH def.
23	1113.8 (11)	0.6	ring distortion
22	1061.7 (48)	1.5	ring distortion
21	1030.5 (111)	18.7	NO str.
20	952.0 (< 1)	0.0	CH o.o.p. def.
19	946.4 (5)	0.0	CH o.o.p. def.
18	876.3 (4)	0.3	CH o.o.p. def.
17	861.8 (7)	0.2	ring distortion
16	800.2 (40)	5.6	ring distortion / NO def.
15	768.7 (76)	0.0	CH o.o.p. def.
14	728.6 (5)	0.4	ring breathing
13	708.1 (17)	8.9	ring distortion
12	675.0 (17)	1.4	ring distortion

11	639.4 (31)	2.6	ring distortion
10	563.6 (2)	0.5	ring distortion
9	554.1 (< 1)	0.0	ring breathing
8	482.9 (4)	2.2	ring distortion
7	442.2 (5)	3.5	ring distortion

Table S3. Experimental and Computed IR frequencies (> 400 cm^{-1}) and intensities (km mol^{-1}) for complex (**3**-AsN).

Mode	$\nu_{theor.}$ B3LYP	$\nu_{exp.}$ Ar-matrix	$\Delta\nu_{cal.}$ $^{15}N/^{14}N$ B3LYP	$\Delta\nu_{exp.}$ $^{15}N/^{14}N$ Ar-matrix	assignment
36	3200.8 (3)	n.o.	0.0	n.o.	A’, CH str.
35	3197.6 (3)	n.o.	0.0	n.o.	A”, CH str.
34	3180.1 (6)	n.o.	0.0	n.o.	A’, CH str.
33	3169.1 (4)	3064.5 (w)	0.0	0.0	A”, CH str.
32	1757.6 (54)	1696.4 (m)	0.0	0.0	A”, C=O asym. str.
31	1728.0 (242)	1668.2/1666.4 (vs)	0.0	0.0	A’, C=O sym. str.
30	1682.1 (< 1)	1626.5 (w)	0.0	0.0	A”, C=C str.
29	1613.4 (3)	1561.8 (w)	0.0	0.0	A’, C=C str.
28	1441.0 (31)	1411.8 (s)	0.0	0.0	A’, CH def.
27	1399.1 (< 1)	n.o.	0.0	n.o.	A”, CH def.
26	1271.5 (52)	1276.5/1267.8 (s)	0.0	0.0	A’, CH def.
25	1191.2 (2)	n.o.	0.0	n.o.	A”, CH def.
24	1159.5 (23)	1141.9 (m)	0.0	0.0	A’, CH def.
23	1134.7 (3)	1120.5 (w)	32.2	33.6	A’, AsN str.
22	1134.3 (12)	1119.4 (w)	0.0	0.0	A”, ring distortion
21	1030.3 (< 1)	n.o.	0.0	n.o.	A”, CH o.o.p. def.
20	1013.6 (< 1)	n.o.	0.0	n.o.	A’, CH o.o.p. def.
19	960.8 (< 1)	n.o.	0.0	n.o.	A’, ring distortion
18	901.6 (< 1)	n.o.	0.0	n.o.	A”, CH o.o.p. def.
17	888.0 (< 1)	n.o.	0.0	n.o.	A”, ring distortion
16	790.8 (< 1)	n.o.	0.0	n.o.	A”, CH o.o.p. def.
15	744.9 (73)	725.8 (vs)	0.0	0.0	A’, CH o.o.p. def.
14	673.6 (3)	n.o.	0.0	n.o.	A’, ring distortion
13	560.1 (14)	552.0 (w)	0.0	n.o.	A”, ring distortion

12	551.9 (< 1)	n.o.	0.0	n.o.	A’, ring distortion
11	473.7 (< 1)	n.o.	0.0	n.o.	A’, ring breathing
10	439.4 (3)	n.o.	0.0	n.o.	A”, ring distortion
9	415.6 (< 1)	n.o.	0.0	n.o.	A”, ring breathing

Table S4. Adiabatic energy gap ($\Delta E_{CS,T}$, kcal mol^{-1}) between singlet closed-shell (CS) and triplet (T) of **2** computed at different DFT and multiconfigurational methods corrected by SOC. [a]ANO-S-VDZP, [b]def2-TZVP. Contributions (%) of double excitations are included at the MS-CASPT2-SOC levels. ≡

Method	$\Delta E_{CS,T}$ / kcal mol^{-1}	%Double
[b]B97-3c	0.61	
[b]M06-2X	3.86	
[b]PBE0	-0.71	
[b]B3LYP-D3	4.59	
[b]ωB97M-V	-0.52	
[a]MS-CASSCF(10,10)	1.42	
[a]MS-CASPT2(10,10)	-6.78	13.21 (7.38)
[a]MS-CASSCF(12,12)	4.65	
[a]MS-CASPT2(12,12)	-11.73	11.78 (8.96)
[b]DLPNO-CCSD(T)	-3.96	

Table S5. Vertical energy gap ($\Delta E_{OS\text{-}GS}$, eV) between singlet open-shell (OS) and the ground state closed -shell (GS = CS) or triplet (GS = T) of **2** computed at different DFT and multiconfigurational methods corrected by SOC. [a]ANO-S-VDZP, [b]def2-TZVP.

Method	$\Delta E_{OS\text{-}CS,}$ / kcal mol^{-1}	$\Delta E_{OS\text{-}T}$ / kcal mol^{-1}
[b]B97-3c	2.61	1.25
[b]M06-2X	3.37	1.48
[b]PBE0	3.09	1.58
[b]B3LYP-D3	2.90	1.46
[a]MS-CASPT2(10,10)	3.71	0.93
[a]MS-CASPT2(12,12)	3.64	0.04

Coordinates and energies

***anti*-(*o*-phenyldioxyl)arseno azide (1a)**
B3LYP/Def2-TZVP

C	-1.26618600	0.69712100	-0.13143700
C	-1.26604000	-0.69675300	-0.13371300
C	-2.37599600	-1.41136500	0.27527600
C	-3.49890600	-0.69694100	0.69229200
C	-3.49906400	0.69413200	0.69453300
C	-2.37631300	1.41015500	0.27983900
H	-2.35914300	-2.49262800	0.26738000
H	-4.37828900	-1.23614300	1.01892600
H	-4.37857000	1.23207600	1.02289900
H	-2.35970500	2.49144100	0.27543600
O	-0.10163000	1.27215300	-0.58787100
O	-0.10138300	-1.27003100	-0.59209800
As	1.15452500	0.00160500	-0.83479900
N	1.80980800	-0.00092900	0.96011700
N	3.02197400	-0.00208000	1.13563800
N	4.12472200	-0.00310000	1.38177900

Zero-point correction= 0.100589 (Hartree/Particle)
Thermal correction to Energy= 0.110743
Thermal correction to Enthalpy= 0.111687
Thermal correction to Gibbs Free Energy= 0.062585
Sum of electronic and zero-point Energies= -2781.763945
Sum of electronic and thermal Energies= -2781.753792
Sum of electronic and thermal Enthalpies= -2781.752847
Sum of electronic and thermal Free Energies= -2781.801949

***syn*-(*o*-phenyldioxyl)arsenoazide (1b)**
B3LYP/Def2-TZVP

C	0.97754800	-0.32270200	0.69701700
C	0.97764700	-0.32378200	-0.69664000
C	2.12097300	-0.01553800	-1.41011900
C	3.27740900	0.29614500	-0.69562700
C	3.27732000	0.29720200	0.69533600
C	2.12079700	-0.01338700	1.41016100
H	2.10354900	-0.02001900	-2.49144500
H	4.18339200	0.53984800	-1.23462300
H	4.18323900	0.54171600	1.23407200
H	2.10324400	-0.01622000	2.49149000
O	-0.22628000	-0.65806200	1.27494500

O	-0.22609400	-0.66012900	-1.27420000
As	-1.50461300	-0.87544600	0.00044200
N	-2.32675400	0.83245900	-0.00076900
N	-1.66058700	1.86425300	-0.00098700
N	-1.12871200	2.85790400	-0.00121700

Zero-point correction= 0.100449 (Hartree/Particle)
Thermal correction to Energy= 0.110632
Thermal correction to Enthalpy= 0.111577
Thermal correction to Gibbs Free Energy= 0.062565
Sum of electronic and zero-point Energies= -2781.765354
Sum of electronic and thermal Energies= -2781.755171
Sum of electronic and thermal Enthalpies= -2781.754227
Sum of electronic and thermal Free Energies= -2781.803238

λ5-(*o*-phenyldioxyl)arseno nitrile (^{1}2)
B3LYP/Def2-TZVP

C	0.00000000	0.69675100	-0.84004700
C	0.00000000	-0.69675100	-0.84004700
C	0.00000000	-1.41201800	-2.02327200
C	0.00000000	-0.69595200	-3.21850700
C	0.00000000	0.69595200	-3.21850700
C	0.00000000	1.41201800	-2.02327200
H	0.00000000	-2.49302500	-2.00500600
H	0.00000000	-1.23416000	-4.15697700
H	0.00000000	1.23416000	-4.15697700
H	0.00000000	2.49302500	-2.00500600
O	0.00000000	1.27936500	0.40548100
O	0.00000000	-1.27936500	0.40548100
As	0.00000000	0.00000000	1.68606100
N	0.00000000	0.00000000	3.31116500

Zero-point correction= 0.090207 (Hartree/Particle)
Thermal correction to Energy= 0.098510
Thermal correction to Enthalpy= 0.099455
Thermal correction to Gibbs Free Energy= 0.056543
Sum of electronic and zero-point Energies= -2672.140719
Sum of electronic and thermal Energies= -2672.132416
Sum of electronic and thermal Enthalpies= -2672.131472
Sum of electronic and thermal Free Energies= -2672.174384

(*o*-phenyldioxyl)arseno nitrene (^{3}2)
B3LYP/Def2-TZVP

C	-0.83033500	-0.72730300	-0.04539700

C	-0.83028800	0.72725200	-0.04553300
C	-2.05357100	1.42647300	0.03445100
C	-3.22076600	0.71118900	0.11275700
C	-3.22082300	-0.71103700	0.11273000
C	-2.05368200	-1.42641900	0.03447100
H	-2.03897100	2.50758700	0.03537000
H	-4.16619200	1.23405300	0.17647900
H	-4.16629400	-1.23382700	0.17640900
H	-2.03917800	-2.50753400	0.03542400
O	0.32333500	-1.29497000	-0.12079600
O	0.32340100	1.29483900	-0.12124200
As	1.88690900	-0.00010200	-0.26360100
N	2.60365000	0.00045900	1.28436700

Zero-point correction= 0.088711 (Hartree/Particle)
Thermal correction to Energy= 0.097647
Thermal correction to Enthalpy= 0.098591
Thermal correction to Gibbs Free Energy= 0.052431
Sum of electronic and zero-point Energies= -2672.149239
Sum of electronic and thermal Energies= -2672.140303
Sum of electronic and thermal Enthalpies= -2672.139359
Sum of electronic and thermal Free Energies= -2672.185519

3-AsN
B3LYP/Def2-TZVP

C	-0.84259800	0.95053100	0.78141000
C	-0.84259800	0.95053100	-0.78141000
C	-0.84527800	2.25542100	-1.45129000
C	-0.84891800	3.38811100	-0.72984000
C	-0.84891800	3.38811100	0.72984000
C	-0.84527800	2.25542100	1.45129000
H	-0.84084800	2.25354100	-2.53360000
H	-0.84893800	4.34896100	-1.23048000
H	-0.84893800	4.34896100	1.23048000
H	-0.84084800	2.25354100	2.53360000
O	-0.84259800	-0.10198900	1.38045000
O	-0.84259800	-0.10198900	-1.38045000
As	0.92769200	-2.38853900	0.00000000
N	2.38412200	-1.69715900	0.00000000

Zero-point correction= 0.088022 (Hartree/Particle)
Thermal correction to Energy= 0.098476
Thermal correction to Enthalpy= 0.099420
Thermal correction to Gibbs Free Energy= 0.047213

Sum of electronic and zero-point Energies= -2672.156888
Sum of electronic and thermal Energies= -2672.146434
Sum of electronic and thermal Enthalpies= -2672.145490
Sum of electronic and thermal Free Energies= -2672.197697

benzo[1,4,2,3]dioxazarsinine (4)
B3LYP/Def2-TZVP

C	0.67436300	-0.63258400	0.19424100
C	0.85953400	0.75104200	0.14894600
C	2.12436000	1.28449500	-0.04820300
H	2.22631100	2.36138700	-0.08059900
C	3.22306000	0.44533300	-0.18546800
C	3.04544500	-0.93205000	-0.15138100
C	1.77372500	-1.46495100	0.02404000
H	4.20886700	0.86951200	-0.32148800
H	3.89300300	-1.59605400	-0.25913800
H	1.60999800	-2.53425400	0.05759800
O	-0.55314800	-1.21576100	0.42162300
O	-0.16284700	1.68957900	0.29842900
N	-1.44612900	1.35254600	-0.07201700
As	-2.00879500	-0.27474700	-0.13774900

Zero-point correction= 0.090971 (Hartree/Particle)
Thermal correction to Energy= 0.098768
Thermal correction to Enthalpy= 0.099712
Thermal correction to Gibbs Free Energy= 0.057182
Sum of electronic and zero-point Energies= -2672.168360
Sum of electronic and thermal Energies= -2672.160563
Sum of electronic and thermal Enthalpies= -2672.159619
Sum of electronic and thermal Free Energies= -2672.202148

Cyclopentadienone (5)
B3LYP/Def2-TZVP

C	-0.08902800	-1.20211300	-0.00032300
C	-1.34392300	-0.75017300	0.00012900
C	-1.34392300	0.75017200	0.00010700
C	-0.08902900	1.20211300	-0.00031200
H	0.26662300	-2.21995000	-0.00071200
H	-2.24704500	-1.34351100	0.00023700
H	-2.24704600	1.34351000	0.00021200
H	0.26662200	2.21995000	-0.00068700
C	0.82183100	0.00000000	0.00053100
O	2.02816000	0.00000000	0.00002000

Zero-point correction= 0.073914 (Hartree/Particle)
Thermal correction to Energy= 0.078624
Thermal correction to Enthalpy= 0.079569
Thermal correction to Gibbs Free Energy= 0.046057
Sum of electronic and zero-point Energies= -268.141370
Sum of electronic and thermal Energies= -268.136660
Sum of electronic and thermal Enthalpies= -268.135715
Sum of electronic and thermal Free Energies= -268.169227

Arsenic mononitride AsN
B3LYP/Def2-TZVP

As	0.00000000	0.00000000	0.28198000
N	0.00000000	0.00000000	-1.32933600

Zero-point correction= 0.002592 (Hartree/Particle)
Thermal correction to Energy= 0.004974
Thermal correction to Enthalpy= 0.005919
Thermal correction to Gibbs Free Energy= -0.019356
Sum of electronic and zero-point Energies= -2290.632430
Sum of electronic and thermal Energies= -2290.630048
Sum of electronic and thermal Enthalpies= -2290.629104
Sum of electronic and thermal Free Energies= -2290.654379

ortho-quinone (3)
B3LYP/Def2-TZVP

C	1.77016400	-0.72980200	0.00002700
C	1.77016900	0.72979100	-0.00002600
C	0.63676700	1.44945300	-0.00004700
C	-0.67398000	0.78222000	0.00001900
C	-0.67398600	-0.78221500	-0.00000200
C	0.63675700	-1.44945600	0.00003900
H	2.73091600	-1.23105700	0.00005200
H	2.73092400	1.23104000	-0.00004500
H	0.63523600	2.53205100	-0.00008800
H	0.63521900	-2.53205400	0.00006300
O	-1.72048400	-1.38756000	-0.00006500
O	-1.72047000	1.38757000	0.00006000

Zero-point correction= 0.084718 (Hartree/Particle)
Thermal correction to Energy= 0.091038
Thermal correction to Enthalpy= 0.091982
Thermal correction to Gibbs Free Energy= 0.053898
Sum of electronic and zero-point Energies= -381.514984
Sum of electronic and thermal Energies= -381.508665

Sum of electronic and thermal Enthalpies= -381.507720
Sum of electronic and thermal Free Energies= -381.545804

N_2
B3LYP/Def2-TZVP

N	0.00000000	0.00000000	-2.218250
N	0.00000000	0.00000000	-3.309152

Zero-point correction= 0.005588 (Hartree/Particle)
Thermal correction to Energy= 0.007948
Thermal correction to Enthalpy= 0.008892
Thermal correction to Gibbs Free Energy= -0.012837
Sum of electronic and zero-point Energies= -109.567674
Sum of electronic and thermal Energies= -109.565314
Sum of electronic and thermal Enthalpies= -109.564369
Sum of electronic and thermal Free Energies= -109.586099

CO
B3LYP/Def2-TZVP

C	0.000000	0.000000	-0.642794
O	0.000000	0.000000	0.482096

Zero-point correction= 0.005045 (Hartree/Particle)
Thermal correction to Energy= 0.007406
Thermal correction to Enthalpy= 0.008350
Thermal correction to Gibbs Free Energy= -0.014072
Sum of electronic and zero-point Energies= -113.357481
Sum of electronic and thermal Energies= -113.355121
Sum of electronic and thermal Enthalpies= -113.354176
Sum of electronic and thermal Free Energies= -113.376598

TS1
B3LYP/Def2-TZVP

C	-1.17220200	-0.66871800	-0.34322500
C	-0.98587400	0.30533400	0.63707200
C	-1.99017900	1.20002800	0.95492300
C	-3.19820000	1.10365900	0.26490800
C	-3.38451600	0.13251700	-0.71345000
C	-2.36817600	-0.76858000	-1.02908500
H	-1.82803400	1.94889800	1.71810100
H	-3.99635400	1.79678600	0.49548800
H	-4.32722000	0.07348400	-1.24120800
H	-2.49652400	-1.52959000	-1.78662200
O	-0.10006400	-1.51333700	-0.53586100

O	0.24602900	0.27549100	1.25882100
As	1.31884100	-0.89698500	0.38998200
N	1.98112100	0.25072600	-0.99290800
N	2.21502300	1.42682300	-0.75451600
N	2.45436000	2.52077200	-0.60482100

Zero-point correction= 0.100449 (Hartree/Particle)
Thermal correction to Energy= 0.109734
Thermal correction to Enthalpy= 0.110678
Thermal correction to Gibbs Free Energy= 0.064587
Sum of electronic and zero-point Energies= -2781.762295
Sum of electronic and thermal Energies= -2781.753010
Sum of electronic and thermal Enthalpies= -2781.752066
Sum of electronic and thermal Free Energies= -2781.798157

TS2
B3LYP/Def2-TZVP

C	1.20031500	-0.08497900	0.69938400
C	1.20044400	-0.08588400	-0.69926800
C	2.31945200	0.31326800	-1.40962700
C	3.43987000	0.73521000	-0.69623400
C	3.43974100	0.73611500	0.69568900
C	2.31919300	0.31509900	1.40942700
H	2.30514100	0.29956100	-2.49096100
H	4.31920900	1.06231200	-1.23526600
H	4.31898100	1.06391800	1.23445600
H	2.30469200	0.30279100	2.49077500
O	0.04460200	-0.52289100	1.28240100
O	0.04484100	-0.52454100	-1.28193200
As	-1.14842700	-1.06931300	0.00047800
N	-2.63449200	-0.18889800	-0.00023200
N	-2.64059000	1.73337200	-0.00109700
N	-3.23628600	2.65055600	-0.00077900

Zero-point correction= 0.096080 (Hartree/Particle)
Thermal correction to Energy= 0.107179
Thermal correction to Enthalpy= 0.108123
Thermal correction to Gibbs Free Energy= 0.056452
Sum of electronic and zero-point Energies= -2781.691600
Sum of electronic and thermal Energies= -2781.680501
Sum of electronic and thermal Enthalpies= -2781.679557
Sum of electronic and thermal Free Energies= -2781.731228

TS3

B3LYP/Def2-TZVP

C	0.88524800	-0.73173500	0.22005100
C	0.88524700	0.73173500	0.22005000
C	2.10038100	1.42675700	-0.00569600
C	3.26283700	0.71260700	-0.10780100
C	3.26283700	-0.71260600	-0.10780100
C	2.10038200	-1.42675700	-0.00569600
H	2.08296200	2.50772600	-0.03100300
H	4.20679200	1.23352300	-0.20332700
H	4.20679300	-1.23352200	-0.20332700
H	2.08296400	-2.50772600	-0.03100300
O	-0.26057600	-1.29124700	0.30425400
O	-0.26057600	1.29124600	0.30425500
N	-3.24840400	0.00000100	0.53690800
As	-1.83797100	0.00000000	-0.28595200

Zero-point correction= 0.088504 (Hartree/Particle)
Thermal correction to Energy= 0.096868
Thermal correction to Enthalpy= 0.097812
Thermal correction to Gibbs Free Energy= 0.053859
Sum of electronic and zero-point Energies= -2672.126187
Sum of electronic and thermal Energies= -2672.117823
Sum of electronic and thermal Enthalpies= -2672.116878
Sum of electronic and thermal Free Energies= -2672.160831

TS4
B3LYP/Def2-TZVP

C	1.17453000	-0.20949100	0.00000000
C	0.00000000	-0.98151600	0.00000000
C	0.02647300	-2.36433100	0.00000000
C	1.27330700	-2.98399600	0.00000000
C	2.45001800	-2.23212500	0.00000000
C	2.41410700	-0.84271200	0.00000000
H	-0.89442500	-2.93109600	0.00000000
H	1.32668600	-4.06479100	0.00000000
H	3.40617200	-2.73912000	0.00000000
H	3.31876700	-0.24934700	0.00000000
O	0.95610600	1.10781300	0.00000000
O	-1.12664800	-0.22726500	0.00000000
As	-0.93786000	1.61636000	0.00000000
N	-2.23876258	0.99387717	-1.15914747

Zero-point correction= 0.088294 (Hartree/Particle)
Thermal correction to Energy= 0.096136

Thermal correction to Enthalpy= 0.097080
Thermal correction to Gibbs Free Energy= 0.054720
Sum of electronic and zero-point Energies= -2672.093114
Sum of electronic and thermal Energies= -2672.085272
Sum of electronic and thermal Enthalpies= -2672.084328
Sum of electronic and thermal Free Energies= -2672.126689

TS5
B3LYP/Def2-TZVP

C	-1.21907100	0.22914500	-0.64270100
C	-1.27921100	-0.47042000	0.56612700
C	-2.40097500	-0.37059800	1.37229600
C	-3.46686800	0.41560100	0.93711700
C	-3.40752400	1.09518100	-0.27601100
C	-2.27347300	1.00956100	-1.08113000
H	-2.43845500	-0.90838400	2.31003100
H	-4.35254700	0.49488300	1.55408400
H	-4.24263800	1.70390300	-0.59637000
H	-2.19802600	1.54232700	-2.01926300
O	-0.05147800	0.08786000	-1.35125300
O	-0.20141800	-1.25424300	0.84110100
As	1.17808300	-0.93604400	-0.35425000
N	1.52346400	0.83603800	-0.30987300
N	3.18607500	1.09043700	0.63653200
N	3.95629300	1.77880900	0.99745500

Zero-point correction= 0.096610 (Hartree/Particle)
Thermal correction to Energy= 0.107316
Thermal correction to Enthalpy= 0.108260
Thermal correction to Gibbs Free Energy= 0.058503
Sum of electronic and zero-point Energies= -2781.684135
Sum of electronic and thermal Energies= -2781.673429
Sum of electronic and thermal Enthalpies= -2781.672484
Sum of electronic and thermal Free Energies= -2781.722241

TS6
B3LYP/Def2-TZVP

C	0.74455700	0.62800300	-0.44275300
C	0.95220000	-0.82122900	-0.37854600
C	2.19713500	-1.31324100	0.14606200
C	3.20056000	-0.44296400	0.44186300
C	2.99524200	0.96497800	0.34322100
C	1.79357900	1.49354800	-0.04324400

H	2.31658900	-2.38564300	0.22523800
H	4.16572200	-0.80975300	0.76703000
H	3.81290600	1.62879000	0.59616200
H	1.62804600	2.56192100	-0.08866200
O	-0.41747600	1.07018800	-0.78466700
O	0.01859700	-1.57839000	-0.74791800
As	-2.06408100	0.28119000	0.22528400
N	-1.70245400	-1.32336100	0.41813300

Zero-point correction= 0.088749 (Hartree/Particle)
Thermal correction to Energy= 0.096891
Thermal correction to Enthalpy= 0.097835
Thermal correction to Gibbs Free Energy= 0.054515
Sum of electronic and zero-point Energies= -2672.132040
Sum of electronic and thermal Energies= -2672.123898
Sum of electronic and thermal Enthalpies= -2672.122954
Sum of electronic and thermal Free Energies= -2672.166274

TS7
B3LYP/Def2-TZVP

C	-1.06094900	-0.39661600	-0.44961900
C	-0.30020600	0.80171000	0.12333600
C	0.98238900	0.98735400	-0.61953700
C	1.87882300	0.00578400	-0.45702600
C	1.40892600	-1.03167100	0.44843400
C	0.14360000	-0.84713500	0.87153600
H	1.06612300	1.82873900	-1.29222300
H	2.82754700	-0.04757200	-0.97203700
H	2.03913900	-1.86235300	0.74731000
H	-0.40974200	-1.43020200	1.59525100
O	-1.97722100	-1.06300600	-0.67949500
O	-1.00260100	1.61236000	0.73186400

Zero-point correction= 0.079203 (Hartree/Particle)
Thermal correction to Energy= 0.086017
Thermal correction to Enthalpy= 0.086961
Thermal correction to Gibbs Free Energy= 0.048223
Sum of electronic and zero-point Energies= -381.416871
Sum of electronic and thermal Energies= -381.410057
Sum of electronic and thermal Enthalpies= -381.409113
Sum of electronic and thermal Free Energies= -381.447850

References

1 F. Neese, *WIREs Comput. Mol. Sci.,* **2022**, *12*, e1606,

2 (a) A. D. Becke, *J. Chem. Phys.* **1993**, *98*, 5648-5652 (b) P. J. Stephens, F. J. Devlin, C. F. Chabalowski, M. J. Frisch, *J. Phys. Chem.* **1994**, *98*, 11623-11627

3 M. J. Frisch, G. W. Trucks, H. B. Schlegel, G. E. Scuseria, M. A. Robb, J. R. Cheeseman, G. Scalmani, V. Barone, B. Mennucci, G. A. Petersson, H. Nakatsuji, M. Caricato, X. Li, H. P. Hratchian, A. F. Izmaylov, J. Bloino, G. Zheng, J. L. Sonnenberg, M. Hada, M. Ehara, K. Toyota, R. Fukuda, J. Hasegawa, M. Ishida, T. Nakajima, Y. Honda, O. Kitao, H. Nakai, T. Vreven, J. A. Montgomery, Jr., J. E. Peralta, F. Ogliaro, M. Bearpark, J. J. Heyd, E. Brothers, K. N. Kudin, V. N. Staroverov, R. Kobayashi, J. Normand, K. Raghavachari, A. Rendell, J. C. Burant, S. S. Iyengar, J. Tomasi, M. Cossi, N. Rega, J. M. Millam, M. Klene, J. E. Knox, J. B. Cross, V. Bakken, C. Adamo, J. Jaramillo, R. Gomperts, R. E. Stratmann, O. Yazyev, A. J. Austin, R. Cammi, C. Pomelli, J. W. Ochterski, R. L. Martin, K. Morokuma, V. G. Zakrzewski, G. A. Voth, P. Salvador, J. J. Dannenberg, S. Dapprich, A. D. Daniels, Ö. Farkas, J. B. Foresman, J. V. Ortiz, J. Cioslowski, D. J. Fox, Gaussian 09, Revision A.2, Gaussian, Inc., Wallingford CT, **2009**.

4 F. Weigend, R. Ahlrichs, *Phys. Chem. Chem. Phys.* **2005**, *7*, 3297–3305.

5 Grimme, J. Antony, S. Ehrlich and H. Krieg, J. Chem. Phys., **2010**, 132, 154104.

6 T. Lu, F. Chen, *J. Comput. Chem.* **2012**, *33*, 580-592.

7 J. G. Brandenburg, C. Bannwarth, A. Hansen, S. Grimme, *J. Chem. Phys*. **2018**, *148*, 064104.

8 Y. Zhao, D. G. Truhlar, *Acc. Chem. Res*. **2008**, *41*, 157-167.

9 C. Adamo, V. Barone, *J. Chem. Phys*. **1999**, *110*, 6158-6170.

10 N. Mardirossian, M. Head-Gordon, J. Chem. Phys. **2016**; 144 (21), 214110.

11 S. Grimme, J. Antony, S. Ehrlich, H. Krieg, *J. Chem. Phys*. **2010**, *132*, 154104.

12 Open Molcas v23.02: G. L. Manni, I. Fdez. Galván, A. Alavi, F. Aleotti, F. Aquilante, J. Autschbach, D. Avagliano, A. Baiardi, J. J. Bao, S. Battaglia, L. Birnoschi, A. Blanco-González, S. I. Bokarev, R. Broer, R. Cacciari, P. B. Calio, R. K. Carlson, R. Carvalho Couto, L. Cerdán, L. F. Chibotaru, N. F. Chilton, J. R. Church, I. Conti, S. Coriani, J. Cuéllar-Zuquin, R. E. Daoud, N. Dattani, P. Decleva, C. de Graaf, M. G. Delcey, L. De Vico, W. Dobrautz, S. S. Dong, R. Feng, N. Ferré, M. Filatov, L. Gagliardi, M. Garavelli, L. González, Y. Guan, M. Guo, M. R. Hennefarth, M. R. Hermes,

C. E. Hoyer, M. Huix-Rotllant, V. K. Jaiswal, A. Kaiser, D. S. Kaliakin, M. Khamesian, D. S. King, V. Kochetov, M. Krośnicki, A. A. Kumaar, E. D. Larsson, S. Lehtola, M.-B. Lepetit, H. Lischka, P. López Ríos, M. Lundberg, D. Ma, S. Mai, P. Marquetand, I. C. D. Merritt, F. Montorsi, M. Mörchen, A. Nenov, V. A. Nguyen, Y. Nishimoto, M. S. Oakley, M. Olivucci, M. Oppel, D. Padula, R. Pandharkar, Q. M. Phung, F. Plasser, G. Raggi, E. Rebolini, M. Reiher, I. Rivalta, D. Roca-Sanjuán, T. Romig, A. A. Safari, A. Sánchez-Mansilla, A. M. Sand, I. Schapiro, T. R. Scott, J. Segarra-Martí, F. Segatta, D.-C. Sergentu, P. Sharma, R. Shepard, Y. Shu, J. K. Staab, T. P. Straatsma, L. K. Sørensen, B. Nunes Cabral Tenorio, D. G. Truhlar, L. Ungur, M. Vacher, V. Veryazov, T. A. Voß, O. Weser, D. Wu, X. Yang, D. Yarkony, C. Zhou, J. P. Zobel, R. Lindh. “The OpenMolcas Web: A Community-Driven Approach to Advancing Computational Chemistry.” *J. Chem. Theory Comp*. **2023**, *19*, 6933–6991.

13 Y. Guo, Ch. Riplinger, U. Becker, D. G. Liakos, Y. Minenkov, L. Cavallo, F. Neese, *J. Chem. Phys.* **2018**, 148 (1), 011101.
